# Effect of acute total sleep deprivation on plasma melatonin, cortisol and metabolite rhythms in females

**DOI:** 10.1111/ejn.14411

**Published:** 2019-05-02

**Authors:** Aya Honma, Victoria L. Revell, Pippa J. Gunn, Sarah K. Davies, Benita Middleton, Florence I. Raynaud, Debra J. Skene

**Affiliations:** ^1^ Chronobiology Faculty of Health and Medical Sciences University of Surrey Guildford UK; ^2^ Surrey Clinical Research Centre University of Surrey Guildford UK; ^3^ Radcliffe Department of Medicine University of Oxford Oxford UK; ^4^ Department of Surgery and Cancer Imperial College London London UK; ^5^ Cancer Research UK Cancer Therapeutics Unit Division of Cancer Therapeutics The Institute of Cancer Research London UK

**Keywords:** circadian rhythms, humans, metabolomics, sleep biomarkers

## Abstract

Disruption to sleep and circadian rhythms can impact on metabolism. The study aimed to investigate the effect of acute sleep deprivation on plasma melatonin, cortisol and metabolites, to increase understanding of the metabolic pathways involved in sleep/wake regulation processes. Twelve healthy young female participants remained in controlled laboratory conditions for ~92 hr with respect to posture, meals and environmental light (18:00–23:00 hr and 07:00‐09:00 hr <8 lux; 23:00–07:00 hr 0 lux (sleep opportunity) or <8 lux (continuous wakefulness); 09:00–18:00 hr ~90 lux). Regular blood samples were collected for 70 hr for plasma melatonin and cortisol, and targeted liquid chromatography–mass spectrometry metabolomics. Timepoints between 00:00 and 06:00 hr for day 1 (baseline sleep), day 2 (sleep deprivation) and day 3 (recovery sleep) were analysed. Cosinor analysis and MetaCycle analysis were performed for detection of rhythmicity. Night‐time melatonin levels were significantly increased during sleep deprivation and returned to baseline levels during recovery sleep. No significant differences were observed in cortisol levels. Of 130 plasma metabolites quantified, 41 metabolites were significantly altered across the study nights, with the majority decreasing during sleep deprivation, most notably phosphatidylcholines. In cosinor analysis, 58 metabolites maintained their rhythmicity across the study days, with the majority showing a phase advance during acute sleep deprivation. This observation differs to that previously reported for males. Our study is the first of metabolic profiling in females during sleep deprivation and recovery sleep, and offers a novel view of human sleep/wake regulation and sex differences.

## Graphical abstract

Young females were kept in controlled laboratory conditions comprising baseline sleep, sleep deprivation and recovery sleep and 1–2 hr blood sampling for 70 hr. Night‐time melatonin increased during sleep deprivation, returning to baseline levels during recovery sleep, whilst no significant changes were observed in cortisol. Of 130 plasma metabolites quantified by targeted metabolomics, 41 were altered across the nights (00:00–06:00 hr) and 58 maintained their rhythmicity across the three study days.

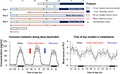

**Abbreviations**
alpha‐AAA: alpha‐Aminoadipic acid
BDI: Beck Depression Index
BMI: body mass index
DLMO: dim light melatonin onset
ESS: Epworth Sleepiness Scale
FDR: false discovery rate
GABA: gamma‐aminobutyric acid
GIT: gastrointestinal tract
LC/MS: liquid chromatography–mass spectrometry
LLOQ: lower limit of quantification
LOD: limit of detection
LOQ: limit of quantification
LXR: liver X receptor
lysoPC: lysophosphatidylcholine
NO: nitric oxide
OPLS‐DA: orthogonal partial least squares discriminant analysis
PC aa: diacylphosphatidylcholine
PC ae: acyl‐alkyl‐phosphatidylcholine
PCA: principal component analysis
PC: principal component
PSQI: Pittsburgh Sleep Quality Index
QC: quality controls
REM: rapid eye movement
REV‐ERBα: reverse‐erb alpha
SCN: suprachiasmatic nuclei
SDMA: symmetric dimethylarginine
SREBP: sterol regulatory element‐binding protein β
t4‐OH‐Pro: trans‐4‐Hydroxyproline

## 1 INTRODUCTION

Sleep comprises approximately one‐third of our lifetime and is an essential physiological function indispensable for survival. In our 24/7 lifestyle of modern society sleep duration has been significantly reduced and more people experience insufficient sleep (Deng et al., 2017). Sleep deprivation has been shown to impact metabolism and immune systems, leading to increased incidence of obesity, impaired glucose tolerance, metabolic syndrome and cardiovascular disease (Huang, Ramsey, Marcheva, & Bass, 2011; Johnston, 2014; Scheer, Hilton, Mantzoros, & Shea, 2009; Spiegel, Knutson, Leproult, Tasali, & Van Cauter, 2005).

Circadian clocks control the timing of most daily biological processes, behaviour and activity, including changes in metabolism and the sleep/wake cycle (Hastings, Reddy, & Maywood, 2003; Mohawk, Green, & Takahashi, 2012). The master clock, located in the hypothalamic suprachiasmatic nuclei (SCN), is entrained to the external light–dark cycle by photic signals transmitted via the retinohypothalamic tract, and coordinates timing of peripheral clocks distributed throughout the body (Barclay et al., 2012; Reppert & Weaver, 2002). Melatonin and cortisol show robust circadian rhythms driven by the SCN oscillator (Czeisler et al., 1999; Gunn, Middleton, Davies, Revell, & Skene, 2016), and melatonin is considered a reliable marker of the master clock (Klerman, Gershengorn, Duffy, & Kronauer, 2002). In entrained conditions, melatonin levels rise in the evening hours, peak in the early hours of the morning and return to basal levels soon after waking (Arendt, 1988).

Liquid chromatography–mass spectrometry (LC/MS) metabolomics analysis has been employed widely to identify and quantify hundreds of metabolites in complex biological matrices such as blood, urine and saliva. Robust circadian rhythms in rodent (Eckel‐Mahan et al., 2012; Minami et al., 2009) and human metabolites have been identified under constant routine conditions (Dallmann, Viola, Tarokh, Cajochen, & Brown, 2012; Kasukawa et al., 2012; Skene et al., 2018). Dallmann et al. (2012) reported that 15% of the metabolites quantified in human plasma and saliva showed circadian variation, particularly amino acids in saliva and fatty acids in plasma. Previously, we reported a significant time‐of‐day variation in 64% of the metabolites measured in healthy young men kept under a controlled light/dark conditions (Davies et al., 2014). In spite of numerous studies on sex differences in metabolic profiling (Mittelstrass et al., 2011; Rist et al., 2017; Ruoppolo et al., 2014), surprisingly little is known about the effect of sleep deprivation on metabolite profiles in females. As the composition of metabolites is different between males and females (Mittelstrass et al., 2011; Pitkanen, Oja, Kemppainen, Seppa, & Mero, 2003; Ruoppolo et al., 2014), the sex‐based differences in metabolite rhythms are of great interest. The aim of this study was to assess the timing, amplitude and phase relationship of the SCN‐driven hormone rhythms (melatonin and cortisol) and plasma metabolite rhythms with a sleep/wake cycle, during 24 hr of wakefulness and a recovery sleep in healthy young females. Assessing the effect of acute sleep deprivation on circulating metabolites and their rhythms may lead to increased understanding of the metabolic pathways involved in sleep/wake regulation and development of sleep biomarkers.

## 2 MATERIALS AND METHODS

### 2.1 Clinical study

Laboratory sessions were conducted at the Surrey Clinical Research Centre (SCRC) at the University of Surrey. Ethics approval for the study was given by the University of Surrey Ethics Committee. All participants provided written informed consent prior to any procedures being performed and participants were allowed to withdraw at any time. All participant information was coded and held in strictest confidence according to the Data Protection Act (UK, 1998). The initial screening phase including eligibility criteria has been reported previously (Ackermann et al., 2012). Briefly, study eligibility was determined by questionnaires including Pittsburgh Sleep Quality Index (PSQI), Epworth Sleepiness Scale (ESS), Horne‐Östberg (H‐O) and Beck Depression Index (BDI), medical and physical assessment, and analysis of blood and urine samples. The participants did not report sleep problems (PSQI score ≤5), depression (BDI <10), daytime sleepiness (ESS <11) and were not extreme morning or evening types (30 < H‐O < 70).

Healthy females (*n* = 12; aged: 25 ± 4 years, mean ± *SD*) with a BMI of 24.9 ± 3.6 kg/m^2^ (mean ± *SD*) were enrolled into the study. All were non‐smokers and unmedicated except that they were taking combined oral contraceptives and were on the active phase during the laboratory session. None had a history of shift work or travel across two or more time zones in the preceding month. Prior to the in‐laboratory session, participants maintained a regular sleep/wake schedule (23:00–07:00 hr) at home for 10 days (confirmed by wrist actigraphy [Actiwatch‐L, Cambridge Neurotechnology Ltd., UK] and sleep diaries). They were also required to have exposure to outdoor light each morning for at least 15 min between 07:00 and 08:30 hr. For 72 hr before the in‐laboratory session, participants were requested to abstain from alcohol, caffeine and strenuous exercise. This baseline‐at‐home period minimised exogenous confounding factors, stabilised circadian phase and ensured participants were not sleep deprived prior to the study.

### 2.2 In‐laboratory session

Participants entered the laboratory at 16:00 hr on day 0 and remained until 12:00 hr on day 4 in a controlled environment with regard to environmental light, sleep and meals, with an adaptation night (day 0) followed by an 8 hr sleep opportunity (23:00 – 07:00 hr) on day 1 (baseline sleep), total sleep deprivation on day 2 (sleep deprivation) and an 8 hr recovery sleep opportunity on day 3 (recovery sleep). Participants remained in <8 lux between 18:00 and 23:00 hr, and between 07:00 and 09:00 hr each day. On days 0, 1 and 3, participants slept between 23:00 and 07:00 hr (0 lux), and remained awake during this time in <8 lux on day 2. Between 09:00 and 18:00 hr each day, participants were free to move about in normal room lighting (~90 lux). The same standardised meals were given on each day of the study at 07:00, 13:00 and 19:00 hr with a snack at 22:00 hr. Blood samples were collected for 70 hr at two hourly intervals for metabolomics analysis and at 1–2 hourly intervals for hormone assays (12:00 hr on day 1–10:00 hr on day 4). Plasma fractions for metabolomics analysis were stored at −80°C until derivatisation; plasma for hormone assays were stored at −20°C until analysis. A detailed scheme of the study protocol is shown in Figure S1.

### 2.3 Sample analysis

Plasma melatonin and cortisol concentrations were measured by radioimmunoassay (Stockgrand Ltd, University of Surrey) as described previously (Sletten, Revell, Middleton, Lederle, & Skene, 2009). Targeted LC/MS was performed on two‐hourly plasma samples to identify and quantify metabolite concentrations, using the AbsoluteIDQ p180 targeted metabolomics kit (Biocrates Life Sciences AG, Innsbruck, Austria), and a Waters Xevo TQ‐S mass spectrometer coupled to an Acquity UPLC system (Waters Corporation, Milford, MA) as previously described (Davies et al., 2014; Isherwood, Van der Veen, Johnston, & Skene, 2017; Skene et al., 2017). The kit provides absolute concentrations of 184 metabolites from six different compound classes (acylcarnitines, amino acids, biogenic amines, lysophosphatidylcholines, glycerophospholipids and sphingolipids). Plasma samples were prepared according to the manufacturer's instructions. Sample order was randomised and three levels of quality controls (QC) were run on each 96‐well plate. Data were normalised between batches using the QC level 2 (QC2) repeats across each plate (*n* = 4) and between plates (*n* = 5) using Biocrates METIDQ software (QC2 correction). Metabolites where >25% concentrations were below the limit of detection (<LOD) or below lower limit of quantification (≪LLOQ) or above limit of quantification (>LOQ) or blank out of range, or the QC2 coefficient of variance was >30%, were excluded (*n* = 54).

### 2.4 Data analysis

Dim light melatonin onset (DLMO) for separate study days was calculated for each participant using the 25% threshold method as previously described (Sletten et al., 2009). Night‐time melatonin levels (00:00–06:00 hr) were compared between the study nights using two‐way repeated ANOVA (R version 6.3.2). Significant *p*‐values were adjusted for multiple comparisons according to the Benjamini–Hochberg false discovery rate (FDR) (Benjamini & Hochberg, 1995). Principal component analysis (PCA) and orthogonal partial least squares discriminant analysis (OPLS‐DA) were performed using SIMCA‐P v.13.0 software (Umetrics, Malmo, Sweden) to visualise the overall variation and components clustering between study days. To examine metabolite levels during the night‐time sleep periods (00:00–06:00 hr), a two‐way repeated ANOVA was carried out (R6.3.2). To assess 24‐hr metabolite rhythmicity, cosinor analysis was performed on the mean *z*‐score values of each study day (12:00–10:00) for each metabolite profile using MATLAB and Bioinformatics Toolbox Release 2016a (The MathWorks, Inc., Natick, Massachusetts, USA). Peak time (acrophase), amplitude and significance of a cosine fit (*p* < 0.05) were determined for each metabolite. Rhythmicity was also assessed using MetaCycle software (Glynn, Chen, & Mushegian, 2006; Hughes, Hogenesch, & Kornacker, 2010) with R6.3.2 which provided acrophase, amplitude and relative amplitude values (FDR adjusted *p* < 0.05). All data are shown as mean ± *SEM*.

## 3 RESULTS

### 3.1 Hormone rhythms

**Figure 1:**
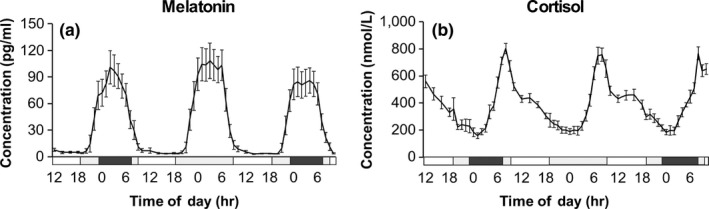
Mean (±SEM) plasma hormone concentrations (*n* = 12) over the 70‐hr sampling protocol (a, melatonin; b, cortisol). The black bar indicates the sleep period, 0 lux, supine; grey bars, wake periods, semi‐recumbent position, <8 lux; white bars, awake and free movement, 90 lux

Night‐time melatonin levels (00:00–06:00 hr) showed a significant increase during sleep deprivation relative to baseline sleep (19% ± 7%), these levels declining back to baseline during recovery sleep (day 1 sleep, 84.8 ± 8.0 pg/ml; day 2 sleep deprivation, 102.1 ± 5.6 pg/ml; day 3 recovery sleep, 81.5 ± 4.2 pg/ml; FDR adjusted *p* < 0.001; Figure 1a). On day 1, the DLMO was 21:56 ± 0:14 hr and this did not change significantly between study days (day 2, 22:02 ± 0:14 hr; day 3, 21:54 ± 0:11 hr). No significant differences were observed in the peak night‐time cortisol levels between the study days (day 1, 268 ± 62 nmol/L; day 2, 309 ± 67 nmol/L; day 3, 287 ± 51 nmol/L; Figure 1b).

### 3.2 Multivariate analysis of metabolites

**Figure 2:**
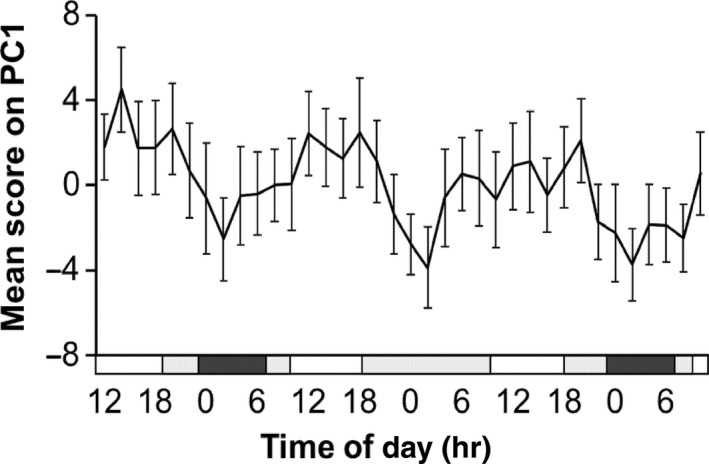
Principal component analysis of all metabolites data was carried out, and the significant time‐of‐day variation in mean score (±SEM) across all subjects on PC1 is shown. The black bar indicates the sleep period, 0 lux, supine; grey bars, wake periods, semi‐recumbent position, <8 lux; white bars, awake and free movement, 90 lux

**Figure 3:**
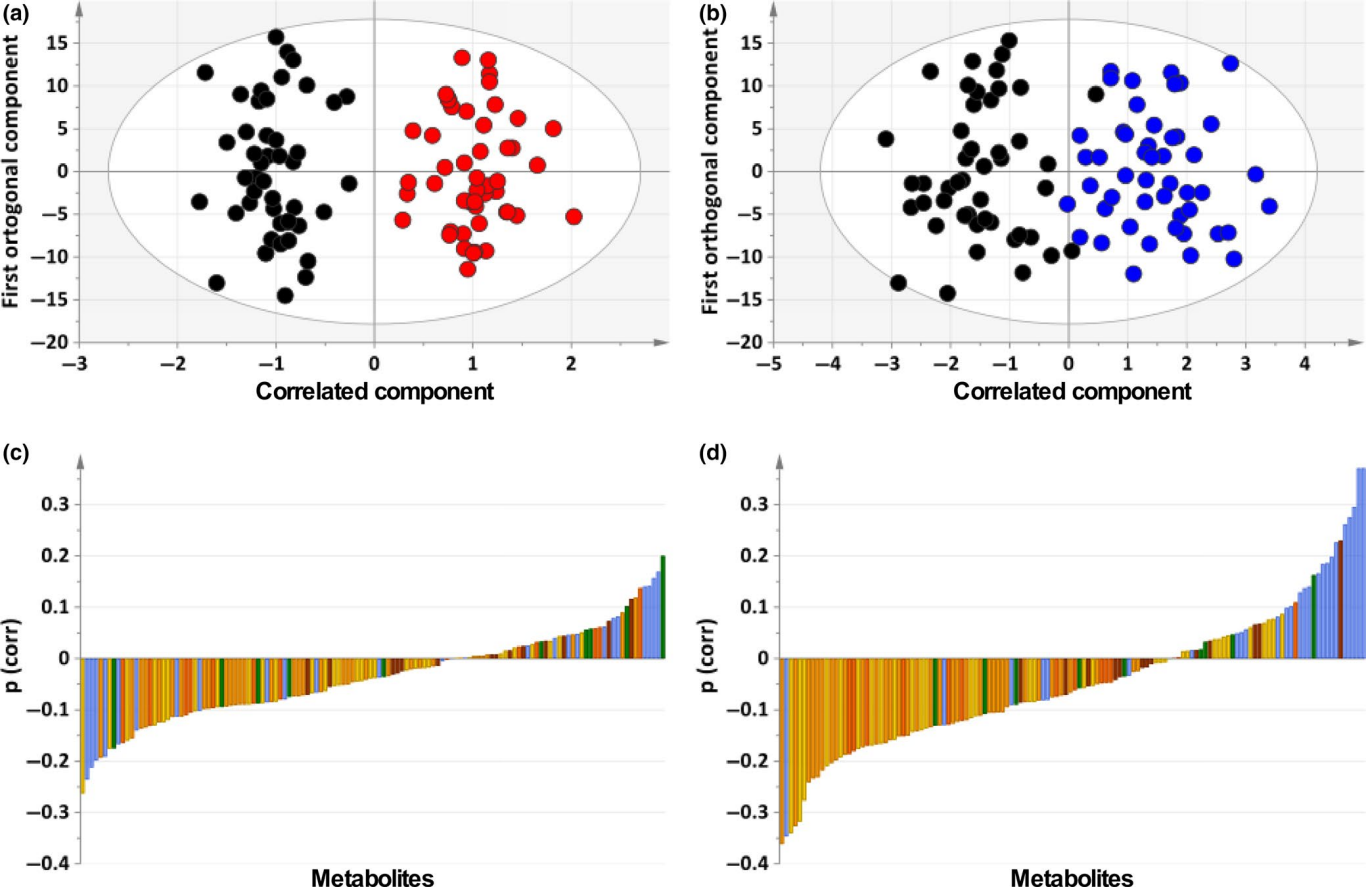
Orthogonal partial least squares discriminant analysis (OPLS‐DA) models of selected time points (00:00–06:00 hr) separated according to sleep status (a, day 1, sleep [black circle] vs. day 2, sleep deprivation [red circle]; b, day 1, sleep (black circle) vs. day 3, recovery sleep [blue circle]). Loading plots for the OPLS‐DA models (c, day 1, sleep vs. day 2, sleep deprivation; d, day 1, sleep vs. day 3, recovery sleep) coloured according to class; amino acids and biogenic amines (blue), acylcarnitines (green), glycerophospholipids (PC aa [yellow], PC ae [light orange], lysoPC [dark orange]) and sphingolipids (brown). Positive *p* (loading) values represent metabolites with higher concentrations, and negative *p* (loading) values represent metabolites with lower concentrations during sleep deprivation (c) and recovery sleep (d) compared with sleep. For lists of metabolites and their corresponding *p* (loading) values, see Table S1

A total of 130 metabolites were detected by targeted LC/MS metabolomics, grouped into amino acids (*n* = 19), acylcarnitines (*n* = 10), biogenic amines (*n* = 7), glycerophospholipids (*n* = 80) and sphingolipids (*n* = 14). Principal component analysis (PCA) of all the data showed there was clear time‐of‐day variation in principal component (PC)1 (amount of variance in the x matrix explained by PC1 (*R* ^2^) = 0.367, estimate of the predictive ability of the model (*Q* ^2^) (cumulative) = 0.358) with the mean score on PC1 having a significant fit to a cosine curve across the study days (acrophase: 15:31, 14:04, 15:20 hr; amplitude: 2.14, 2.18, 1.99, day1, 2, 3, respectively) (Figure 2). OPLS‐DA models, validated by permutation analysis and a CV ANOVA *p*‐value of the model, showed clear separation between sleep (00:00–06:00 hr, day 1) and sleep deprivation (00:00–06:00 hr, day 2); *Q* ^2^ (cumulative) = 0.641, *R* ^2^ *X* (cumulative) = 0.707, *R* ^2^ *Y* (cumulative) = 0.903 (Figure 3a), between sleep (00:00–06:00 hr, day 1) and recovery sleep (00:00–06:00 hr, day 3); *Q* ^2^ (cumulative) = 0.608, *R* ^2^ *X* (cumulative) = 0.601, *R* ^2^ *Y* (cumulative) = 0.790 (Figure 3b), and between sleep deprivation (00:00–06:00 hr, day 2) and recovery sleep (00:00–06:00 hr, day 3); *Q* ^2^ (cumulative) = 0.439, *R* ^2^ *X* (cumulative) = 0.592, *R* ^2^ *Y* (cumulative) = 0.722 (Figure S2a). The p(corr) loading plot for the OPLS‐DA models are shown in Figure 3c,d and Figure S2b; metabolites with negative values decreased in sleep deprivation and metabolites with positive values increased in sleep deprivation. Plasma levels of histidine, glutamate, glutamine, lysine, carnitine, symmetric dimethylarginine (SDMA) and six glycerophospholipids (lysoPC a C18:0, PC aa C36:5, PC aa C40:3, PC aa C42:2, PC ae C36:3 and PC ae C42:2) decreased during sleep deprivation and tetradecenoylcarnitine, taurine, methionine levels increased during sleep deprivation (|*p*(corr)| >0.15) (Table S1).

### 3.3 Univariate analysis of metabolites

Of the 130 metabolites, 41 (32%) metabolites showed significant differences in their night‐time concentrations (0:00–6:00 hr) in the study days (FDR adjusted *p* < 0.05); 12 (of 19) amino acids (threonine, glycine, alanine, glutamate, glutamine, histidine, lysine, asparagine, ornithine, methionine, arginine and proline), carnitine, two biogenic amines (taurine, SDMA), 25 (of 80) glycerophospholipids (lysoPC a C18:0, PC aa C36:1, PC aa C36:5, PC aa C38:0, PC aa C38:5, PC aa C40:2, PC aa C40:3, PC aa C40:4, PC aa C40:5, PC aa C40:6, PC aa C42:2, PC aa C42:6, PC ae C34:2, PC ae C34:3, PC ae C36:0, PC ae C36:3, PC ae C36:4, PC ae C36:5, PC ae C38:0, PC ae C38:5, PC ae C38:6, PC ae C40:1, PC ae C42:1, PC ae C42:2 and PC ae C42:4) and 1 (of 14) sphingolipid (SM C20:2) (Table S2). Only 15 out of 130 metabolites (12%) were significantly different between the sleep and sleep deprivation periods, 5 amino acids (histidine, glutamate, threonine, lysine, citrulline), carnitine, SDMA, lysoPC a C18:0 and 7 phosphatidylcholines. Except threonine, all of these metabolites exhibited decreased levels during sleep deprivation compared to baseline sleep. Of these, seven metabolites showed a significant difference between sleep deprivation and recovery sleep; histidine, lysine, citrulline, carnitine and SDMA returned to baseline levels, two phosphatidylcholines (PC aa C36:5 and PC ae C42:2) showed a further decrease and threonine showed a further increase during recovery sleep (Table S3).

### 3.4 Daily rhythms in metabolites

**Figure 4:**
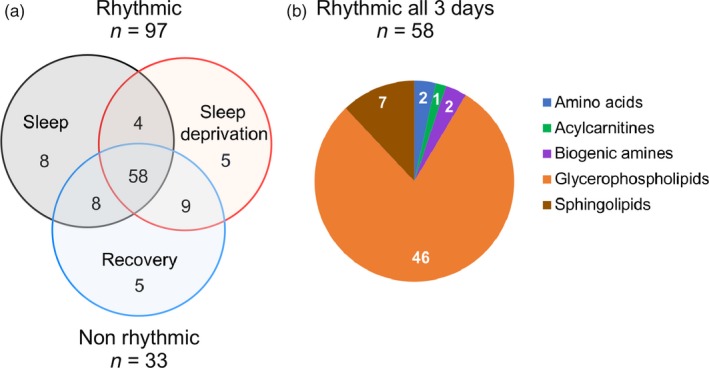
Metabolites with a significant cosine rhythm during the three study days. (a) Venn diagram showing the number of metabolites exhibiting a significant fit to a cosine curve on day 1 (sleep, grey circle), day 2 (sleep deprivation, red circle), and day 3 (recovery sleep, blue circle). (b) Pie charts showing the proportion of metabolites from each metabolite class exhibiting a significant fit to a cosine curve on all study days

To assess rhythmic variation of the metabolites, cosinor analysis of the mean *z*‐score metabolite profiles on each study day was performed. Of the 130 metabolites, 33 (25%) metabolites showed no rhythmicity across any of the study days (Figure 4a). Of the 97 remaining metabolites, significant daily rhythms were observed in 78 (60%) metabolites on day 1 (normal sleep/wake); three amino acids (asparagine, glutamate, glycine), valerylcarnitine, four biogenic amines (alpha‐Aminoadipic acid [alpha‐AAA], kynurenine, SDMA, trans‐4‐Hydroxyproline [t4‐OH‐Pro]), 60 glycerophospholipids and 10 sphingolipids, with most metabolites (*n* = 63; 81%) peaking during the day (06:00–18:00 hr). Of these, 62 (79%) metabolites maintained their rhythmicity during sleep deprivation, with 58 (74%) maintaining their rhythmicity across all three study days. Metabolites (*n* = 58) showing daily rhythms across all three study days included two amino acids (asparagine, glutamate), one acylcarnitine (valerylcarnitine), two biogenic amines (SDMA, t4‐OH‐Pro), 46 glycerophospholipids and seven sphingolipids (Figure 4b). Of these, the majority (*n* = 56; 97%), except valerylarnitine and SDMA, showed an advanced acrophase during sleep deprivation (−1.6 ± 0.1 hr), with the majority (*n* = 49; 88%) subsequently returning to baseline timing during recovery sleep (Table S4). Metabolite data were also analysed with MetaCycle (JTK‐CYCLE). Using this analysis, significant daily rhythms were observed in only 18% (*n* = 23) of metabolites on day 1 (normal sleep/wake); namely two amino acids (glutamate, glycine), two biogenic amines (SDMA, t4‐OH‐Pro) and 19 glycerophospholipids. Of these, 12 (52%) metabolites maintained rhythmicity on day 2 (sleep deprivation) and only 8 (35%) metabolites maintained rhythmicity across the three study days; t4‐OH‐Pro and seven glycerophospholipids (Figure S3, Table S5).

### 3.5 Sex differences

**Figure 5:**
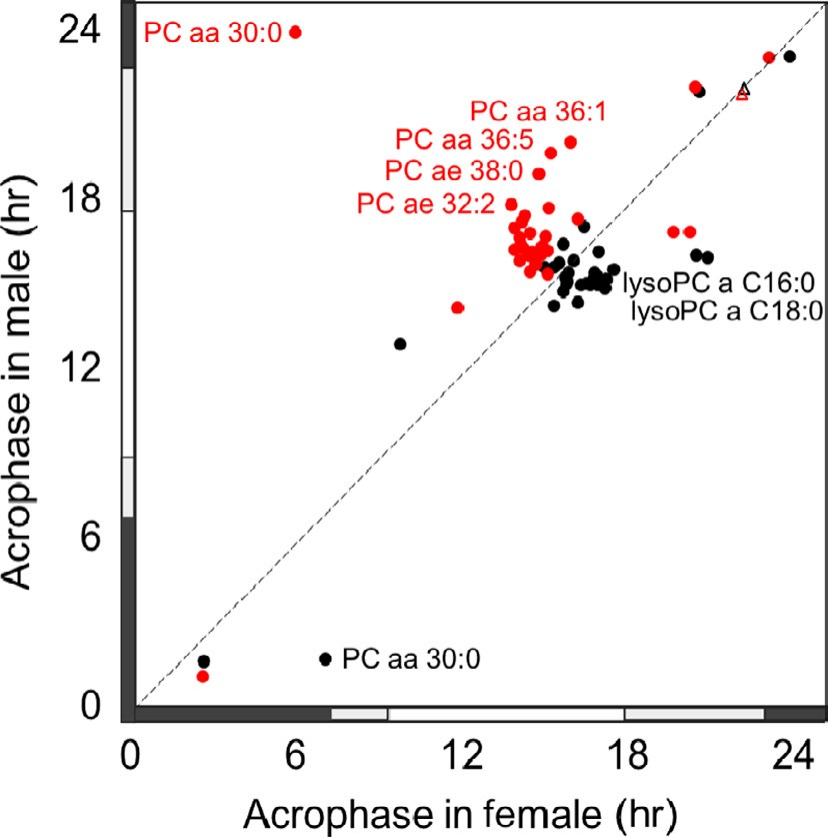
Peak times (acrophase) of 31 common metabolites with a significant fit to a cosine curve in both males and females on day 1 (black circle) and day 2 (red circle). Dim light melatonin onset times on both days (day 1: black triangle, day 2: red triangle) are shown. Labelled metabolites had a difference in acrophase time of 4 hr or more between males and females. For the details of metabolite acrophase times, see Table S7

In our previous study, diurnal rhythms in plasma metabolites in young males were also observed (Davies et al., 2014). Reanalysing these data using the same criteria as for the female data as described in the Methods above (Isherwood et al., 2017; Skene et al., 2017), 141 metabolites were quantified. Of these, 77 (55%) exhibited a daily rhythm that had a significant fit to a cosine curve on day 1 (sleep) with most (*n* = 53; 69%) maintaining their 24 hr rhythmicity on day 2 (sleep deprivation). Of these, the majority (*n* = 45; 85%) showed a delay in acrophase time during sleep deprivation (1.4 ± 0.2 hr) (Table S6). The mean score on PC1 had a significant fit to a cosine curve on day 1 with an acrophase of 14:47 hr, but no significant fit on day 2 (15:50 hr, *p* = 0.053) (Figure S4). In a subset analysis of both sexes, there were 32 common metabolites that exhibited diurnal rhythms on day 1 and day 2. The mean acrophase time of these metabolites was significantly later in the female group on day 1 (15:48 ± 0:40 hr) compared to the male group (14:53 ± 0:42 hr, *p* < 0.001, Student's *T*‐test), and earlier on day 2 (14:29 ± 0:38 hr) compared to males (16:52 ± 0:38 hr, *p* < 0.001, Student's *T*‐test) (Figure 5, Table S7), although no significant difference in DLMO (female: 21:56 ± 0:14 hr, male: 21:58 ± 0:31 hr) was observed. Metabolites that were only rhythmic on day 1 and day 2 in females (*n* = 13) and only rhythmic in males (*n* = 15) are also presented in Table S7.

Sex differences in the metabolites affected by sleep deprivation were also observed (Table S8). In females, 15 (12%) of the 130 metabolites were significantly altered during sleep deprivation, all but threonine showing decreased levels. By contrast, in the males, 37 out of 141 metabolites (26%) exhibited significant changes during sleep deprivation, all increasing during sleep deprivation.

## 4 DISCUSSION

This study aimed to assess the effect of a single night of total sleep deprivation followed by recovery sleep under controlled laboratory conditions on both biomarkers of the central circadian pacemaker and plasma metabolic profiles in young females.

The pineal hormone melatonin showed significantly increased plasma concentrations during sleep deprivation, which is in agreement with our previous study in young males (Ackermann et al., 2013; Davies et al., 2014), and returned to baseline levels during a subsequent night of recovery sleep. Although the mechanism underlying this increase is not known, we previously showed induction of a heat shock protein coding gene HSPA1B expression during sleep deprivation (Ackermann et al., 2012), suggesting a direct consequence of the oxidative stress induced by sleep loss, which may lead to up‐regulation of melatonin, a well‐known antioxidant (Carrillo‐Vico et al., 2005; Reiter, Tan, Osuna, & Gitto, 2000).

Increased melatonin levels during sleep deprivation might also reflect increased adrenergic stimulation (Klein & Weller, 1973; Skene, Bojkowski, & Arendt, 1994). Sleep deprivation may also act directly on SCN neuronal activity reducing SCN output (Deboer, Detari, & Meijer, 2007) that, in turn, may activate melatonin synthesis via the SCN‐pineal multisynaptic pathway. While we assume that the increased plasma levels arise from the pineal gland, there is also the possibility that the melatonin is derived from the gastrointestinal tract (GIT). The amount of melatonin in the GIT is estimated to be more than 400 times that of the pineal gland (Huether, 1993). There is some evidence that food deprivation and fasting increases melatonin levels in the brain and GIT of mice (Bubenik, Ball, & Pang, 1992) and in the serum of humans (Beitins et al., 1985). It can be speculated that the increased energy expenditure and fasting during sleep deprivation may induce melatonin release from the GIT into the circulation.

In our study, only 15 metabolites levels were significantly different between the sleep and sleep deprivation periods: histidine, glutamate, threonine, lysine, citrulline, carnitine, SDMA, lysoPC a C18:0 and seven phosphatidylcholines. Apart from threonine, all of these metabolites exhibited decreased levels during sleep deprivation. Both during the sleep period and during sleep deprivation, the participants were in a fasting state. Thus, the metabolites that changed during sleep deprivation were not a direct effect of fasting. However, the interaction of increased energy consumption during the sleep deprivation period and the fasting state may contribute to the observed metabolite differences.

l‐Histidine is an essential amino acid that is a precursor to histamine. Being able to cross the blood–brain barrier (Hargreaves & Pardridge, 1988), histidine levels in the blood may reflect brain histamine levels and histaminergic neuronal activity in the tuberomammillary nucleus, where they play a critical role in the maintenance of arousal (Saper, Fuller, Pedersen, Lu, & Scammell, 2010). The observed reduction of blood histidine levels during sleep deprivation may reflect its increased degradation into histamine as an acute response to sleep loss, returning to baseline levels during recovery sleep. Glutamate is the most abundant excitatory neurotransmitter in the vertebrate nervous system (Meldrum, 2000) and also serves as the precursor for the synthesis of the inhibitory gamma‐aminobutyric acid (GABA) in GABAergic neurons, which plays an important role in the sleep‐promoting systems in the brain (Gallopin et al., 2000; Sherin, Shiromani, McCarley, & Saper, 1996). Glutamine is the most abundant free amino acid in blood, making a large contribution to cellular respiration as an energy source following glucose and lactate (Hui et al., 2017). Glutamate and glutamine play important roles in removing excess nitrogen, detoxifying ammonia in the skeletal muscle, brain, kidney and liver (Adeva, Souto, Blanco, & Donapetry, 2012). In our study, plasma glutamate decreased during sleep deprivation and did not return to baseline levels during recovery sleep. Glutamine also showed a trend to decrease during sleep deprivation, returning to baseline levels during recovery sleep. These results can be attributed to increased energy demands and increased need for removal of metabolic waste products from the brain during sleep deprivation (Xie et al., 2013). Diurnal changes in brain glutamate and glutamine levels were recently observed in healthy young adults with a significant overnight reduction assessed by proton magnetic resonance spectroscopy (Volk, Jaramillo, Merki, O'Gorman Tuura, & Huber, 2018). In addition, a previous study reported reduced glutamine levels associated with lower total sleep time and increased wake‐time after sleep onset (Miller et al., 2017), supporting our hypothesis.

Carnitine plays an important role in energy production by conjugating fatty acids for transport into the mitochondria, by forming a long chain acetylcarnitine ester (Rebouche, 2004). Several reports have indicated a role for fatty acid β‐oxidation and the carnitine system in sleep/wake regulation. However, the role of carnitine and acylcarnitine in the brain remains unknown. Fasting in systemic carnitine‐deficient juvenile visceral steatosis (*jvs* ^−/−^) mice exhibited a higher frequency of fragmented wakefulness and rapid eye movement sleep, and reduced locomotor activity (Yoshida et al., 2006). In these mice, a lower percentage of c‐Fos‐positive orexin neurons, which regulate the sleep/wake cycle and food intake, and reduced orexin‐A concentration in the cerebrospinal fluid was observed (Yoshida et al., 2006). It has been reported that supplementation of acetylcarnitine produces releasable glutamate (Tanaka et al., 2003; Toth, Harsing, Sershen, Ramacci, & Lajtha, 1993). Orexin‐producing cells in the lateral hypothalamus are mainly innervated by excitatory neurons containing glutamate (Horvath & Gao, 2005; Li, Gao, Sakurai, & van den Pol, 2002). These findings indicate that acylcarnitine availability is essential for normal sleep/wake regulation and orexin cell functions. Thus, our results suggest increased acylcarnitine consumption during sleep deprivation to maintain wakefulness via the orexinergic arousal system.

Previous studies have indicated that cholesterol/lipid metabolism is regulated by the sleep/wake cycle and that sleep deprivation may modify it (Jones, Pfister‐Genskow, Benca, & Cirelli, 2008; Moller‐Levet et al., 2013). Detailed characterisation of lipid profiles, however, has remained unclear. In our previous study using LC/MS analysis, increases in blood glycerophospholipids levels were observed during the acute sleep deprivation period (Davies et al., 2014). Elevated phospholipids were also reported after sleep deprivation in the rat and human (Weljie et al., 2015). Another study with MS‐based lipidomic analysis showed increased levels of lipids following sleep restriction (five nights of 4 hr/night in bed) compared to controls (8 hr in bed) (Aho et al., 2016). These authors reported that sleep loss decreased the expression of genes encoding cholesterol transporters by activating inflammatory responses (Aho et al., 2016). Toll‐like receptors suppress the activity of liver X receptor (LXR) regulating cholesterol metabolism (Castrillo et al., 2003; Choi et al., 2015), which leads to decreased reverse cholesterol transport and synthesis of fatty acids and triglycerides (Joseph, Castrillo, Laffitte, Mangelsdorf, & Tontonoz, 2003; Lee & Tontonoz, 2015). These findings suggest that sleep deprivation/restriction modifies cholesterol pathways at the level of gene expression and serum lipoproteins.

Symmetric dimethylarginine is reported to be an endogenous marker of renal function (Kielstein, Salpeter, Bode‐Boeger, Cooke, & Fliser, 2006) and is negatively associated with nitric oxide (NO) production (Bode‐Boger et al., 2006). As NO is reported to regulate the sleep/wake state via inactivation of orexin neurons (Cespuglio, Amrouni, Meiller, Buguet, & Gautier‐Sauvigne, 2012; Yamakawa, Kurauchi, Hisatsune, Seki, & Katsuki, 2018), a reduction in plasma SDMA could be related to the disruption of sleep homeostasis during the study.

Previous studies have suggested that amino acid and fatty acid metabolism is under circadian control (Blanco et al., 2007; Bray & Young, 2011; Huang et al., 2011). Reverse‐erb alpha (REV‐ERBα), a circadian transcription gene encoding the nuclear orphan receptor, regulates hepatic gluconeogenesis, adipocyte differentiation and lipid metabolism (Duez & Staels, 2008). REV‐ERBα also controls oscillations in sterol regulatory element‐binding protein (SREBP) activity, through modulation of insulin‐induced gene 2, a resident protein of the endoplasmic reticulum, and thereby in the daily expression of SREBP target genes involved in cholesterol and lipid metabolism (Le Martelot et al., 2009). These authors reported that alteration of oxysterol synthesis and LXR activity mediated the effects of REV‐ERBα on bile acid metabolism.

Metabolomics and lipidomics studies have analysed plasma samples from participants kept under constant routine laboratory conditions and found that 15% of all identified metabolites, most notably fatty acids (Dallmann et al., 2012) and 13% of lipid metabolites (Chua et al., 2013) were under circadian clock control suggesting a direct effect of circadian timing on fatty acid metabolism. We ourselves have seen 33% rhythmic glycerophospholipids under constant routine conditions following a simulated day shift (Skene et al., 2018) supporting this hypothesis. In the current study, of the 130 metabolites identified, the largest group of compounds exhibiting diurnal variation across the three study days, was lipids (*n* = 46 with cosinor analysis and *n* = 7 with MetaCycle analysis), implying a robust circadian timing system underlying the daily rhythms in lipid metabolites. The peak phases of rhythmic phosphatidylcholines and sphingolipids mostly occurred in the daytime (15:35 ± 0:14 hr, *n* = 60) which is consistent with previous reports (Ang et al., 2012; Dallmann et al., 2012; Davies et al., 2014). Interestingly, most metabolites (*n* = 56, 97% with cosinor analysis and *n* = 6, 75% with MetaCycle analysis) maintaining rhythmicity across the three study days showed a tendency to phase advance during the 24 hr of wakefulness, most notably the glycerophospholipids, with the majority returning to baseline timings during the subsequent recovery sleep. Besides the circadian timing system, the behavioural state (e.g. feeding/fasting, sleep/wakefulness, postural changes) may affect the 24 hr rhythmicity of metabolites. Currently, we are unable to say which of these behaviours contribute most to the observed metabolite rhythmicity. Further systematic experiments are needed to accurately assess the contribution of each.

Recently, sleep restriction was reported to affect circadian rhythmicity of the rodent and human transcriptome (Barclay et al., 2012; Moller‐Levet et al., 2013). Employing transcriptome analysis of human blood, Moller‐Levet et al. (2013) reported that the number of genes whose transcripts had circadian expression were reduced in sleep restriction. Moreover, during the sleep restriction condition, those genes with peak times during the biological night had later peak times, and genes with peak times in the biological day had earlier peak times. The peak phases of rhythmic metabolites likely relate to the physiologic pathways in which they are involved. For example, the fat clock promotes lipogenesis and adiponectin production during the wake period (Bass & Takahashi, 2010), which is consistent with our data of most phospholipids peaking during the day.

We performed two distinct analyses for assessing the daily rhythmicity of metabolites: cosinor analysis and MetaCycle. The methods showed large differences in the number of rhythmic metabolites, one possible reason being that MetaCycle performed FDR correction of the p‐values. Thus, the data analysed using MetaCycle were more strictly selected, comprising a subset of metabolites which also showed statistical significance with cosinor analysis.

To our knowledge, this is the first report of metabolic profiling during acute total sleep deprivation and recovery sleep conditions in females. Taking data from our previous study of healthy young males (Davies et al., 2014) and reanalysing with the same criteria as the current female study, 37 out of 141 (26%) detected metabolites exhibited significant changes during sleep deprivation compared with during sleep, including tryptophan, serotonin, taurine, 6 acylcarnitines, 24 glycerophospholipids and 4 sphingolipids. All of these metabolites exhibited increased levels during sleep deprivation. By contrast, in the current female study, only 12% (*n* = 15) of the 130 identified metabolites exhibited significant changes and most of these (*n* = 14, 93%) exhibited decreased levels during sleep deprivation. Although the same study protocol, research facilities and analytical methodology was used, the differences between the two studies are striking. Although previous studies have suggested that men and women, and male and female rodents respond differently to sleep deprivation (Acheson, Richards, & de Wit, 2007; Baratta et al., 2018; Ferrara et al., 2015), until now its effect on circulating metabolite profiles have not been investigated.

Sex differences in plasma metabolites have been reported with most studies showing higher levels of amino acids and acylcarnitine in males compared to females (Mittelstrass et al., 2011; Pitkanen et al., 2003; Ruoppolo et al., 2014). In addition, the use of oral contraceptives, as used in the current study, induces specific alterations in the serum metabolic status (Ruoppolo et al., 2014). The levels of phosphatidylcholines and sphingolipids show a tendency to be higher in females, supporting an increase in lipid storage rather than metabolism in females (Mittelstrass et al., 2011; Rist et al., 2017).

Sleep disruption can affect peripheral clocks, where each tissue keeps metabolic processes in synchrony (Bass & Takahashi, 2010). As the utilisation of energy and metabolic rate are different between genders (Arciero, Goran, & Poehlman, 1993; Volpi, Lucidi, Bolli, Santeusanio, & De Feo, 1998), the timing of metabolite rhythms and their alternation during sleep deprivation may vary between the sexes.

Dim light melatonin onset has been reported to be significantly earlier in females than males (Cain et al., 2010; Mongrain, Lavoie, Selmaoui, Paquet, & Dumont, 2004; Van Reen et al., 2013), but there was no difference observed in our study. Gunn et al. (2016) demonstrated females exhibited significantly higher levels of plasma melatonin and cortisol than males during a constant routine protocol. Our previous male study also demonstrated increased night‐time melatonin levels during sleep deprivation (Davies et al., 2014), but the increase was greater in males than females (27 ± 5%, 19 ± 7% respectively), suggesting a sex difference in reaction to sleep loss/wakefulness. In addition, alteration in blood melatonin levels can affect metabolic profiles (Bass & Takahashi, 2010; Dubocovich & Markowska, 2005), which may relate to different trends (increase/decrease) in metabolite concentrations during sleep deprivation between males and females. Female rodents are reported to have shorter circadian periods than males (Davis, Darrow, & Menaker, 1983; Schull et al., 1989), and in a human study under laboratory conditions to detect intrinsic circadian period (Duffy et al., 2011), females indeed had a shorter period (tau). Our data showed the mean acrophase of common metabolites occurred significantly later in females, which then advanced to earlier than that of males during sleep deprivation. This tendency was also observed in the acrophase of the mean score on PC1 of all metabolites, with an advance during sleep deprivation in females but a delay in males (Figure 2 and Figure S4).

In the current study, the effect of the menstrual cycle was controlled for by only including women on hormonal contraceptives. In follow‐up studies, it will be important to investigate how these metabolites vary across the menstrual cycle (follicular and luteal phases) and whether this is affected by sleep deprivation.

In conclusion, in this first report of metabolic profiling during sleep deprivation and recovery sleep in females, we have identified a panel of plasma metabolites that are significantly altered during acute sleep deprivation and recovery sleep. There appears to be some differences between males and females in the response to sleep deprivation but, as yet, the underlying basis of these differences cannot be fully explained. These findings will be useful in guiding the design and interpretation of future metabolite‐based studies from the point of sex differences.

## ACKNOWLEDGEMENTS

This work was funded by a UK Biotechnology and Biological Sciences Research Council (BBSRC) Grant (BB/I019405/1). Melatonin and cortisol measurements were carried out by Stockgrand Ltd. The staff of the Surrey Clinical Research Centre (SCRC) and the Metabolomics Core Facility at the University of Surrey in particular Chris Mitchell, is acknowledged. The authors also thank Dr Anne Skeldon (Department of Mathematics, University of Surrey) and Dr Jeroen Pennings (National Institute for Public Health and the Environment, Bilthoven, The Netherlands) for assistance and writing the analysis scripts for MatLab and R respectively.

## COMPETING INTERESTS

Debra J. Skene and Benita Middleton are codirectors of Stockgrand Ltd.

## AUTHOR CONTRIBUTIONS

AH: data analysis, writing manuscript; VLR: experimental design, data collection, reviewing manuscript; PJG: data analysis, reviewing manuscript; SKD: data collection, data analysis, reviewing manuscript; BM: data analysis, reviewing manuscript; FIR: experimental design, data analysis, reviewing manuscript; DJS: experimental design, data analysis, writing manuscript.

## Data Availability

The data sets analysed during the current study are available from the corresponding author (at d.skene@surrey.ac.uk) on reasonable request.
