# Supplementary material for: Effect of acute total sleep deprivation on plasma melatonin, cortisol and metabolite rhythms in females
Source: Eur J Neurosci. 2019 May 2;51(1):366–78. doi: 10.1111/ejn.14411 (PMC7027445; doi:10.1111/ejn.14411)
Supplement: Supplementary file 1 [file EJN-51-366-s001.pdf]

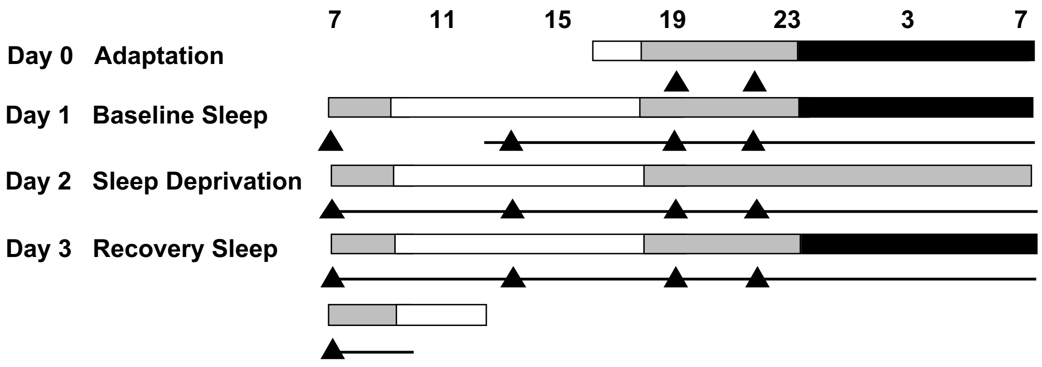

Figure S1. Study protocol. Black bars indicate sleep periods, supine, 0 lux; grey bars, wake periods, semi-recumbent position, <8 lux; white bars, awake and free movement, 90 lux; (▲) standardized meals; (—) blood sampling period across 70 h.

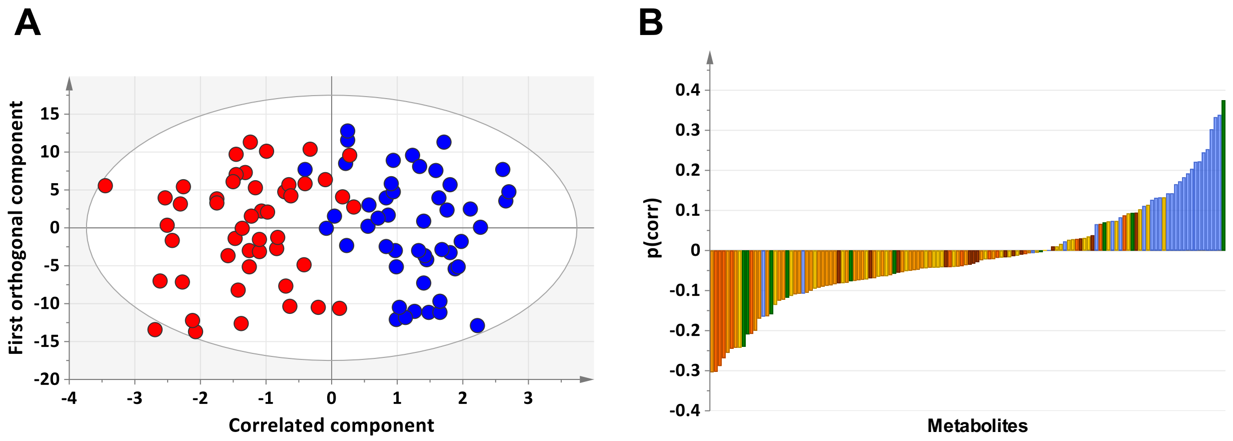

Figure S2. OPLS-DA models separated day 2 (sleep deprivation) and day 3 (recovery sleep) for selected time points (00:00–06:00 h). (A) Score plot of sleep deprivation ● vs. recovery sleep ●. (B) Loading plot for the OPLS-DA models, coloured according to class; amino acids and biogenic amines (blue), acylcarnitines (green), glycerophospholipids (PC aa (yellow), PC ae (light orange), lysoPC (dark orange)) and sphingolipids (brown). Positive P (loading) values represent metabolites with higher concentrations, and negative P (loading) values represent metabolites with lower concentrations during recovery sleep compared with sleep deprivation.

Supplementary Table S1. OPLS-DA loadings (p(corr)) of the metabolites in models, comparing sleep vs. sleep deprivation (A), sleep vs. recovery sleep (B), and sleep deprivation vs. recovery sleep (C).

A. sleep vs. sleep deprivation

Metabolite	p(corr)	Metabolite	p(corr)	Metabolite	p(corr)
PC aa C40:3	-0.263	lysoPC a C20:4	-0.080	PC ae C34:1	0.006
Histidine	-0.236	Citrulline	-0.080	PC ae C32:2	0.006
Glutamate	-0.212	Octadecanoylcarnitine	-0.075	PC ae C30:2	0.008
Glutamine	-0.199	PC ae C34:3	-0.074	SM C16:0	0.008
PC ae C42:2	-0.194	PC ae C38:0	-0.073	SM (OH) C14:1	0.009
Lysine	-0.192	PC ae C42:1	-0.072	PC aa C42:4	0.010
PC aa C36:5	-0.175	SM C26:0	-0.070	PC aa C34:2	0.016
Carnitine	-0.175	PC aa C36:3	-0.068	SM (OH) C24:1	0.016
SDMA	-0.167	Arginine	-0.066	PC aa C32:0	0.021
lysoPC a C18:0	-0.164	PC ae C40:2	-0.066	lysoPC a C26:1	0.023
PC aa C42:2	-0.160	PC aa C38:4	-0.063	SM C26:1	0.024
PC ae C36:3	-0.156	SM C24:1	-0.055	Glycine	0.025
alpha-AAA	-0.140	PC aa C42:5	-0.053	PC aa C34:3	0.028
PC ae C38:5	-0.137	PC aa C38:6	-0.052	lysoPC a C24:0	0.032
PC ae C44:6	-0.134	PC ae C42:5	-0.052	Octadecenoylcarnitine	0.033
lysoPC a C16:0	-0.131	PC ae C40:4	-0.051	SM C18:1	0.034
PC aa C36:1	-0.130	PC ae C40:3	-0.046	PC aa C34:4	0.034
PC ae C40:1	-0.125	PC aa C40:6	-0.046	Serotonin	0.040
PC aa C38:5	-0.124	PC aa C42:0	-0.044	PC aa C32:1	0.044
PC aa C40:2	-0.120	PC ae C40:6	-0.041	SM (OH) C16:1	0.044
PC ae C42:3	-0.114	PC aa C28:1	-0.039	Alanine	0.046
Ornithine	-0.114	PC aa C38:3	-0.038	PC ae C34:0	0.047
PC ae C44:5	-0.113	t4-OH-Pro	-0.037	Tyrosine	0.048
lysoPC a C20:3	-0.110	Valerylcarnitine	-0.036	PC aa C30:0	0.052
lysoPC a C18:2	-0.105	PC ae C32:1	-0.034	Propionylcarnitine	0.057
PC aa C42:6	-0.103	SM (OH) C22:2	-0.031	Hexadecanoylcarnitine	0.058
ADMA	-0.102	SM (OH) C22:1	-0.029	lysoPC a C28:0	0.059
PC ae C44:4	-0.098	SM C16:1	-0.024	lysoPC a C28:1	0.061
PC ae C38:6	-0.097	PC aa C40:4	-0.023	Leucine	0.062
lysoPC a C18:1	-0.096	PC ae C36:1	-0.021	SM C18:0	0.073
PC aa C38:0	-0.094	PC aa C36:6	-0.020	Kynurenine	0.079
Butyrylcarnitine	-0.094	PC ae C36:2	-0.018	Threonine	0.083
PC ae C36:4	-0.093	PC aa C36:4	-0.018	PC ae C36:0	0.090
PC ae C42:4	-0.092	PC aa C34:1	-0.017	Acetylcarnitine	0.102
PC ae C34:2	-0.090	PC ae C38:4	-0.015	SM C20:2	0.116
PC ae C40:5	-0.089	SM C24:0	-0.014	PC ae C38:2	0.119
PC ae C36:5	-0.089	Asparagine	-0.005	lysoPC a C26:0	0.137
PC aa C36:2	-0.089	lysoPC a C16:1	-0.003	Isoleucine	0.140
lysoPC a C17:0	-0.088	Proline	-0.001	Tryptophan	0.141
Octadecadienylcarnitine	-0.088	PC aa C36:0	0.001	Methionine	0.157
PC aa C40:5	-0.087	PC ae C30:0	0.002	Taurine	0.170
Valine	-0.086	Phenylalanine	0.002	Tetradecenoylcarnitine	0.200
PC ae C44:3	-0.085	PC aa C32:3	0.002		
PC aa C42:1	-0.084	PC ae C38:3	0.005		

B. sleep vs. recovery sleep

Metabolite	p(corr)	Metabolite	p(corr)	Metabolite	p(corr)
PC ae C42:2	-0.361	PC ae C40:6	-0.111	lysoPC a C20:3	0.003
Glutamate	-0.347	Octadecadienylcarnitine	-0.108	PC aa C34:3	0.015
PC aa C40:2	-0.341	PC ae C38:2	-0.106	PC aa C36:6	0.015
PC ae C34:3	-0.327	PC ae C36:0	-0.106	Kynurenine	0.017
PC aa C42:2	-0.318	PC ae C44:4	-0.105	SM (OH) C16:1	0.017
PC aa C40:3	-0.277	PC ae C40:3	-0.104	Valerylcarnitine	0.018
PC ae C36:3	-0.241	PC ae C40:4	-0.094	Butyrylcarnitine	0.032
PC ae C36:5	-0.234	Valine	-0.090	SM (OH) C24:1	0.035
PC ae C40:1	-0.231	Octadecanoylcarnitine	-0.090	PC aa C36:3	0.038
PC ae C38:5	-0.218	SM (OH) C14:1	-0.086	PC aa C36:2	0.038
PC aa C36:5	-0.209	PC ae C40:2	-0.085	PC aa C32:3	0.043
PC ae C34:2	-0.203	PC aa C42:4	-0.085	PC aa C30:0	0.046
PC ae C38:6	-0.199	PC aa C38:4	-0.085	Propionylcarnitine	0.047
PC aa C36:1	-0.193	PC ae C38:4	-0.082	Histidine	0.049
PC ae C32:2	-0.188	Citrulline	-0.082	Tryptophan	0.052
lysoPC a C24:0	-0.186	SDMA	-0.081	Lysine	0.057
lysoPC a C26:1	-0.180	PC ae C36:1	-0.075	PC aa C34:2	0.061
PC aa C42:6	-0.176	lysoPC a C17:0	-0.074	SM C18:1	0.066
PC ae C36:4	-0.172	PC ae C38:0	-0.072	SM C18:0	0.067
lysoPC a C28:0	-0.169	SM (OH) C22:2	-0.071	PC aa C34:4	0.069
PC aa C42:5	-0.169	PC ae C30:0	-0.066	PC aa C32:1	0.076
PC ae C44:6	-0.167	lysoPC a C18:1	-0.061	PC aa C36:4	0.077
PC aa C42:1	-0.166	Hexadecanoylcarnitine	-0.057	Leucine	0.082
PC ae C40:5	-0.165	PC aa C40:6	-0.057	PC aa C34:1	0.087
PC ae C42:4	-0.159	SM C16:1	-0.053	t4-OH-Pro	0.099
PC aa C38:5	-0.158	PC aa C38:3	-0.053	Phenylalanine	0.102
PC ae C30:2	-0.152	PC ae C38:3	-0.050	lysoPC a C16:1	0.109
lysoPC a C18:0	-0.151	lysoPC a C26:0	-0.049	Arginine	0.129
PC ae C42:5	-0.150	lysoPC a C20:4	-0.047	Glutamine	0.137
PC aa C40:5	-0.143	lysoPC a C16:0	-0.047	Ornithine	0.140
PC ae C44:3	-0.141	SM C16:0	-0.041	Carnitine	0.163
PC aa C38:0	-0.140	SM C24:1	-0.036	Glycine	0.166
PC aa C42:0	-0.136	Octadecenoylcarnitine	-0.035	Proline	0.184
PC ae C42:3	-0.134	ADMA	-0.034	Tyrosine	0.186
Acetylcarnitine	-0.131	PC ae C34:1	-0.026	Asparagine	0.198
PC ae C44:5	-0.130	PC ae C34:0	-0.024	Isoleucine	0.227
alpha-AAA	-0.130	SM (OH) C22:1	-0.018	SM C20:2	0.230
lysoPC a C28:1	-0.129	SM C24:0	-0.017	Serotonin	0.262
PC ae C32:1	-0.126	SM C26:1	-0.011	Threonine	0.276
PC ae C42:1	-0.123	PC aa C32:0	-0.009	Taurine	0.296
lysoPC a C18:2	-0.121	PC aa C36:0	-0.009	Methionine	0.371
PC ae C36:2	-0.120	PC aa C38:6	-0.007	Alanine	0.371
PC aa C28:1	-0.117	Tetradecenoylcarnitine	0.000		
PC aa C40:4	-0.112	SM C26:0	0.001		

C. sleep deprivation vs. recovery sleep

Metabolite	p(corr)	Metabolite	p(corr)	Metabolite	p(corr)
PC ae C38:2	-0.304	PC aa C42:5	-0.064	PC aa C34:2	0.017
lysoPC a C28:0	-0.303	PC ae C32:1	-0.062	Valine	0.023
lysoPC a C26:1	-0.289	Octadecadienylcarnitine	-0.058	PC aa C36:6	0.027
lysoPC a C26:0	-0.269	SM (OH) C14:1	-0.055	PC aa C38:6	0.028
PC ae C34:3	-0.256	PC ae C40:2	-0.054	lysoPC a C16:0	0.029
lysoPC a C24:0	-0.245	PC aa C40:3	-0.052	SM C18:1	0.030
PC ae C36:0	-0.243	PC ae C38:3	-0.051	PC aa C34:4	0.031
PC aa C40:2	-0.243	PC ae C36:4	-0.049	PC aa C32:3	0.035
Acetylcarnitine	-0.240	PC ae C38:4	-0.048	SM C26:0	0.038
Tetradecenoylcarnitine	-0.210	PC ae C30:0	-0.045	Citrulline	0.066
lysoPC a C28:1	-0.208	PC aa C28:1	-0.045	lysoPC a C20:3	0.067
PC ae C30:2	-0.200	PC ae C36:1	-0.043	Valerylcarnitine	0.070
PC ae C42:2	-0.170	PC aa C40:4	-0.043	PC aa C36:0	0.073
Glutamate	-0.165	PC aa C38:5	-0.043	Isoleucine	0.074
PC ae C32:2	-0.164	PC aa C40:5	-0.042	PC aa C32:1	0.074
Hexadecanoylcarnitine	-0.159	SM C24:1	-0.042	alpha-AAA	0.083
PC aa C42:0	-0.136	lysoPC a C18:0	-0.041	lysoPC a C16:1	0.088
PC ae C36:2	-0.125	PC aa C36:5	-0.041	PC aa C36:2	0.093
PC ae C40:1	-0.123	PC ae C34:1	-0.040	Butyrylcarnitine	0.094
Octadecenoylcarnitine	-0.118	lysoPC a C18:2	-0.039	SM C20:2	0.095
PC aa C42:2	-0.114	PC ae C44:6	-0.037	PC aa C34:1	0.103
PC ae C34:2	-0.110	SM (OH) C16:1	-0.036	t4-OH-Pro	0.111
PC ae C36:5	-0.108	SM (OH) C24:1	-0.033	PC aa C36:4	0.114
Tryptophan	-0.108	SM (OH) C22:2	-0.029	Phenylalanine	0.127
PC ae C42:5	-0.106	PC ae C38:0	-0.025	ADMA	0.132
PC ae C44:3	-0.101	PC aa C38:0	-0.022	Serotonin	0.133
PC aa C42:1	-0.095	lysoPC a C17:0	-0.022	PC aa C36:3	0.133
PC ae C36:3	-0.093	PC ae C40:4	-0.021	Taurine	0.143
PC ae C38:6	-0.090	lysoPC a C18:1	-0.019	Tyrosine	0.143
PC ae C38:5	-0.088	PC aa C32:0	-0.018	Glycine	0.166
PC ae C40:5	-0.087	SM C16:1	-0.017	SDMA	0.173
PC ae C40:6	-0.084	PC aa C38:4	-0.017	Proline	0.183
SM C26:1	-0.082	SM C24:0	-0.014	Ornithine	0.192
PC aa C42:6	-0.082	PC aa C40:6	-0.013	Arginine	0.204
PC ae C42:3	-0.081	SM C18:0	-0.010	Methionine	0.222
Octadecanoylcarnitine	-0.077	lysoPC a C20:4	-0.009	Asparagine	0.222
PC ae C40:3	-0.076	PC ae C44:4	-0.007	Threonine	0.245
PC ae C42:1	-0.075	Kynurenine	-0.007	Lysine	0.253
PC ae C34:0	-0.073	PC aa C34:3	-0.006	Histidine	0.303
PC aa C36:1	-0.072	Propionylcarnitine	-0.004	Alanine	0.333
SM C16:0	-0.070	PC aa C30:0	0.002	Glutamine	0.339
PC ae C44:5	-0.069	Leucine	0.002	Carnitine	0.375
PC aa C42:4	-0.066	SM (OH) C22:1	0.010		
PC ae C42:4	-0.064	PC aa C38:3	0.011		

Supplementary Table S2. Night time metabolite concentrations (μM, mean ± SEM, 0:00 – 6:00 h) showing significant differences in the 3 days (day1: sleep, day 2: sleep deprivation, day 3: recovery sleep).

Metabolite	Day 1			Day 2			Day 3			
	mean ± SEM (μM)			mean ± SEM (μM)			mean ± SEM (μM)			FDR P
Alanine	272.6	±	9.4	276.1	±	10.2	313.5	±	10.5	0.000
Arginine	76.5	±	2.4	73.8	±	2.9	79.5	±	2.5	0.015
Asparagine	55.3	±	1.3	55.0	±	1.7	58.6	±	1.5	0.002
Glutamine	570.0	±	10.6	546.7	±	9.9	589.6	±	8.8	0.000
Glutamate	44.5	±	1.5	39.9	±	1.6	37.7	±	1.6	0.000
Glycine	177.5	±	6.2	179.1	±	7.8	200.3	±	9.1	0.000
Histidine	101.0	±	1.7	95.8	±	2.0	101.3	±	1.7	0.002
Lysine	250.6	±	6.8	232.6	±	7.3	251.4	±	7.4	0.002
Methionine	21.9	±	0.5	23.1	±	0.6	23.9	±	0.5	0.004
Ornithine	40.5	±	1.3	38.7	±	1.7	42.9	±	1.6	0.003
Proline	151.2	±	6.5	150.5	±	6.0	159.7	±	6.1	0.041
Threonine	152.0	±	6.3	157.9	±	5.4	175.4	±	5.8	0.000
Carnitine	24.9	±	0.9	23.1	±	0.7	26.3	±	0.8	0.000
SDMA	0.42	±	0.0	0.39	±	0.0	0.41	±	0.0	0.017
Taurine	46.5	±	1.2	49.1	±	1.1	50.8	±	1.4	0.002
lysoPC a C18:0	11.3	±	0.4	10.5	±	0.4	10.6	±	0.4	0.003
PC aa C36:1	41.1	±	1.3	39.1	±	1.1	37.9	±	1.0	0.022
PC aa C36:5	16.4	±	1.1	14.0	±	0.8	13.2	±	0.8	0.000
PC aa C38:0	2.3	±	0.1	2.2	±	0.1	2.1	±	0.1	0.002
PC aa C38:5	37.5	±	1.5	35.1	±	1.2	34.4	±	1.1	0.001
PC aa C40:2	0.21	±	0.0	0.20	±	0.0	0.18	±	0.0	0.001
PC aa C40:3	0.50	±	0.0	0.46	±	0.0	0.46	±	0.0	0.005
PC aa C40:4	2.5	±	0.1	2.5	±	0.1	2.4	±	0.1	0.049
PC aa C40:5	6.1	±	0.3	5.8	±	0.2	5.6	±	0.2	0.005
PC aa C40:6	22.5	±	1.2	21.6	±	1.0	21.4	±	1.0	0.046
PC aa C42:2	0.15	±	0.0	0.14	±	0.0	0.14	±	0.0	0.029
PC aa C42:6	0.39	±	0.0	0.37	±	0.0	0.36	±	0.0	0.004
PC ae C34:2	8.8	±	0.3	8.6	±	0.2	8.3	±	0.2	0.010
PC ae C34:3	5.4	±	0.1	5.2	±	0.1	4.8	±	0.1	0.000
PC ae C36:0	0.66	±	0.0	0.69	±	0.0	0.63	±	0.0	0.011
PC ae C36:3	5.5	±	0.2	5.2	±	0.1	5.0	±	0.1	0.000
PC ae C36:4	11.3	±	0.4	10.8	±	0.4	10.5	±	0.4	0.004
PC ae C36:5	8.3	±	0.3	7.9	±	0.3	7.4	±	0.3	0.000
PC ae C38:0	1.44	±	0.1	1.38	±	0.1	1.35	±	0.1	0.050
PC ae C38:5	11.3	±	0.3	10.9	±	0.3	10.6	±	0.3	0.008
PC ae C38:6	5.2	±	0.2	5.0	±	0.2	4.7	±	0.2	0.000
PC ae C40:1	0.72	±	0.0	0.67	±	0.0	0.63	±	0.0	0.000
PC ae C42:1	0.24	±	0.0	0.23	±	0.0	0.22	±	0.0	0.028
PC ae C42:2	0.38	±	0.0	0.36	±	0.0	0.34	±	0.0	0.000
PC ae C42:4	0.74	±	0.0	0.72	±	0.0	0.70	±	0.0	0.049
SM C20:2	0.89	±	0.0	0.93	±	0.0	0.98	±	0.0	0.001

Supplementary Table S3. Night time metabolite concentrations (µM, mean ± SEM, 0:00 – 6:00 h) showing significant differences between day 1 (sleep) vs. day 2 (sleep deprivation), day 2 vs. day 3 (recovery sleep) and day 1 vs. day 3.

Metabolite	Day 1 mean ± SEM (µM)			Day 2 mean ± SEM (µM)			FDR P
Citrulline	24.3	±	0.9	22.8	±	1.1	0.038
Glutamate	44.5	±	1.5	39.9	±	1.6	0.009
Histidine	101.0	±	1.7	95.8	±	2.0	0.004
Lysine	250.6	±	6.8	232.6	±	7.3	0.005
Threonine	152.0	±	6.3	157.9	±	5.4	0.045
Carnitine	24.9	±	0.9	23.1	±	0.7	0.038
SDMA	0.42	±	0.0	0.39	±	0.0	0.000
lysoPC a C18:0	11.3	±	0.4	10.5	±	0.4	0.002
PC aa C36:5	16.4	±	1.1	14.0	±	0.8	0.000
PC aa C38:0	2.3	±	0.1	2.2	±	0.1	0.041
PC aa C38:5	37.5	±	1.5	35.1	±	1.2	0.008
PC aa C40:3	0.50	±	0.0	0.46	±	0.0	0.008
PC aa C40:5	6.1	±	0.3	5.8	±	0.2	0.045
PC ae C36:3	5.5	±	0.2	5.2	±	0.1	0.019
PC ae C42:2	0.38	±	0.0	0.36	±	0.0	0.029

Metabolite	Day 2 mean ± SEM (µM)			Day 3 mean ± SEM (µM)			FDR P
Alanine	276.1	±	10.2	313.5	±	10.5	0.000
Arginine	73.8	±	2.9	79.5	±	2.5	0.002
Asparagine	55.0	±	1.7	58.6	±	1.5	0.002
Glutamine	546.7	±	9.9	589.6	±	8.8	0.000
Glycine	179.1	±	7.8	200.3	±	9.1	0.000
Histidine	95.8	±	2.0	101.3	±	1.7	0.004
Lysine	232.6	±	7.3	251.4	±	7.4	0.002
Ornithine	38.7	±	1.7	42.9	±	1.6	0.001
Proline	150.5	±	6.0	159.7	±	6.1	0.033
Threonine	157.9	±	5.4	175.4	±	5.8	0.000
Tryptophan	59.77	±	1.48	56.64	±	1.3	0.030
Carnitine	23.1	±	0.7	26.3	±	0.8	0.002
Acetylcarnitine	3.97	±	0.29	3.32	±	0.2	0.029
SDMA	0.39	±	0.0	0.41	±	0.0	0.006
PC aa C36:5	14.0	±	0.8	13.2	±	0.8	0.006
PC aa C40:2	0.20	±	0.0	0.18	±	0.0	0.044
PC ae C34:3	5.2	±	0.1	4.8	±	0.1	0.000
PC ae C36:0	0.69	±	0.0	0.63	±	0.0	0.008
PC ae C36:5	7.9	±	0.3	7.4	±	0.3	0.006
PC ae C38:6	5.0	±	0.2	4.7	±	0.2	0.014
PC ae C40:1	0.67	±	0.0	0.63	±	0.0	0.041
PC ae C42:2	0.36	±	0.0	0.34	±	0.0	0.035

Metabolite	Day 1			Day 3			
	mean ± SEM (μM)			mean ± SEM (μM)			FDR P
Alanine	272.6	±	9.4	313.5	±	10.5	0.000
Asparagine	55.3	±	1.3	58.6	±	1.5	0.002
Glutamate	44.5	±	1.5	37.7	±	1.6	0.000
Glycine	177.5	±	6.2	200.3	±	9.1	0.000
Methionine	21.9	±	0.5	23.9	±	0.5	0.001
Threonine	152.0	±	6.3	175.4	±	5.8	0.000
Taurine	46.5	±	1.2	50.8	±	1.4	0.018
lysoPC a C18:0	11.3	±	0.4	10.6	±	0.4	0.012
PC aa C36:1	41.1	±	1.3	37.9	±	1.0	0.016
PC aa C36:5	16.4	±	1.1	13.2	±	0.8	0.000
PC aa C38:0	2.3	±	0.1	2.1	±	0.1	0.000
PC aa C38:5	37.5	±	1.5	34.4	±	1.1	0.001
PC aa C40:2	0.21	±	0.0	0.18	±	0.0	0.000
PC aa C40:3	0.50	±	0.0	0.46	±	0.0	0.004
PC aa C40:4	2.5	±	0.1	2.4	±	0.1	0.018
PC aa C40:5	6.1	±	0.3	5.6	±	0.2	0.004
PC aa C40:6	22.5	±	1.2	21.4	±	1.0	0.026
PC aa C42:2	0.15	±	0.0	0.14	±	0.0	0.020
PC aa C42:6	0.39	±	0.0	0.36	±	0.0	0.001
PC ae C34:2	8.8	±	0.3	8.3	±	0.2	0.004
PC ae C34:3	5.4	±	0.1	4.8	±	0.1	0.000
PC ae C36:3	5.5	±	0.2	5.0	±	0.1	0.000
PC ae C36:4	11.3	±	0.4	10.5	±	0.4	0.002
PC ae C36:5	8.3	±	0.3	7.4	±	0.3	0.000
PC ae C38:0	1.44	±	0.1	1.35	±	0.1	0.038
PC ae C38:5	11.3	±	0.3	10.6	±	0.3	0.004
PC ae C38:6	5.2	±	0.2	4.7	±	0.2	0.000
PC ae C40:1	0.72	±	0.0	0.63	±	0.0	0.000
PC ae C40:5	2.84	±	0.1	2.69	±	0.1	0.041
PC ae C42:1	0.24	±	0.0	0.22	±	0.0	0.030
PC ae C42:2	0.38	±	0.0	0.34	±	0.0	0.000
PC ae C42:4	0.74	±	0.0	0.70	±	0.0	0.031
PC ae C44:6	0.98	±	0.0	0.94	±	0.0	0.045
SM C20:2	0.89	±	0.0	0.98	±	0.0	0.000

Supplementary Table S4. Metabolic profiling data: cosinor analysis (amplitude, acrophase) for each measured metabolite on 3 days (day1: sleep, day 2: sleep deprivation, day 3: recovery sleep) and the change in phase between each day.

Metabolite	Day 1			Day 2			Day 3			Phase shift Δ φ (dec.h)		
	AMP	phase (dec. h)	p value	AMP	phase (dec. h)	p value	AMP	phase (dec. h)	p value	D1 vs. D2	D1 vs. D3	D2 vs. D3
Alanine			0.48			0.20			0.50			
Arginine			0.68			0.46			0.63			
Asparagine	0.81	23.04	0.04	0.81	21.50	0.04	0.85	22.3	0.02	-1.54	-0.74	0.81
Citrulline			0.13			0.29			0.12			
Glutamine			0.27			0.33			0.58			
Glutamate	0.84	2.46	0.00	0.80	2.32	0.00	0.66	2.9	0.02	-0.14	0.41	0.55
Glycine	0.82	2.20	0.02			0.14	0.41	1.4	0.02		-0.83	
Histidine			0.32			0.43			0.30			
Isoleucine			0.79			0.79			0.77			
Leucine			0.91			0.90			0.96			
Lysine			0.62			0.26			0.34			
Methionine			0.82			0.59			0.57			
Ornithine			0.48			0.28			0.56			
Phenylalanine			0.11			0.07			0.06			
Proline			0.17			0.10			0.14			
Threonine			0.21			0.13	0.83	2.4	0.02			
Tryptophan			0.14			0.45	1.00	18.4	0.05			
Tyrosine			0.43			0.21			0.51			
Valine			0.69			0.22			0.25			
Carnitine			0.13			0.73	0.33	5.0	0.04			
Acetylcarnitine			0.64			0.83			0.70			
Propionylcarnitine			0.58			0.56			0.92			
Butyrylcarnitine			0.11			0.17			0.18			
Valerylcarnitine	0.47	14.03	0.00	0.47	16.04	0.03	0.41	16.9	0.00	2.01	2.88	0.87
Tetradecenoylcarnitine			0.09			0.48			0.21			
Hexadecanoylcarnitine			0.47			0.65			0.59			
Octadecanoylcarnitine			0.60			0.45			0.57			
Octadecenoylcarnitine			0.50			0.63			0.63			
Octadecadienylcarnitine			0.20			0.19			0.14			
ADMA			0.35			0.79			0.68			
alpha-AAA	0.40	23.41	0.04	0.72	22.55	0.00			0.11	-0.87		
Kynurenine	0.90	16.45	0.04			0.21	0.52	14.4	0.00		-2.04	
SDMA	0.75	9.56	0.00	0.88	11.55	0.00	0.68	9.1	0.02	1.99	-0.44	-2.43
Serotonin			0.46			0.10			0.38			
t4-OH-Pro	1.07	1.88	0.00	1.10	1.26	0.00	1.16	1.8	0.00	-0.62	-0.05	0.56
Taurine			0.10	0.49	18.39	0.01			0.08			
lysoPC a C16:0	0.76	20.30	0.00	0.82	19.40	0.00	0.81	20.1	0.00	-0.90	-0.19	0.71
lysoPC a C16:1	0.91	18.90	0.00	0.73	18.46	0.00	0.76	18.6	0.00	-0.45	-0.26	0.18
lysoPC a C17:0	0.64	20.48	0.01	0.74	19.71	0.00	0.68	19.8	0.01	-0.77	-0.68	0.09
lysoPC a C18:0	0.70	20.67	0.01	0.67	19.97	0.00	0.68	21.1	0.01	-0.71	0.42	1.13
lysoPC a C18:1	0.88	20.54	0.00	0.93	20.38	0.00	0.87	20.3	0.00	-0.16	-0.23	-0.07
lysoPC a C18:2	1.07	20.38	0.00	1.09	20.09	0.00	1.07	19.8	0.00	-0.29	-0.61	-0.31
lysoPC a C20:3	0.94	20.02	0.00	0.89	19.55	0.00	0.81	19.6	0.01	-0.48	-0.41	0.06
lysoPC a C20:4	0.91	20.44	0.00	0.92	19.89	0.00	0.90	19.5	0.00	-0.54	-0.90	-0.36

Metabolite	Day 1			Day 2			Day 3			Phase shift Δ φ (dec.h)		
	AMP	phase (dec. h)	p value	AMP	phase (dec. h)	p value	AMP	phase (dec. h)	p value	D1 vs. D2	D1 vs. D3	D2 vs. D3
lysoPC a C24:0			0.49			0.64			0.05			
lysoPC a C26:0	0.41	14.75	0.05			0.49			0.09			
lysoPC a C26:1			0.15			0.93			0.16			
lysoPC a C28:0			0.35			0.85			0.16			
lysoPC a C28:1	0.43	14.80	0.01			0.54			0.13			
PC aa C28:1	0.40	16.54	0.01	0.37	14.40	0.03	0.44	15.7	0.01	-2.15	-0.81	1.34
PC aa C30:0	0.56	6.81	0.01	0.76	5.58	0.00	0.79	6.2	0.00	-1.24	-0.62	0.62
PC aa C32:0			0.16			0.15			0.21			
PC aa C32:1	0.76	10.86	0.00	0.94	10.06	0.00	0.87	9.9	0.00	-0.80	-0.92	-0.13
PC aa C32:3			0.59	0.50	11.39	0.03			0.05			
PC aa C34:1			0.20			0.16			0.37			
PC aa C34:2			0.46			0.42			0.55			
PC aa C34:3	0.60	13.55	0.01			0.08			0.05			
PC aa C34:4	0.45	10.77	0.03			0.07	0.51	9.4	0.04		-1.38	
PC aa C36:0	0.31	16.42	0.01			0.15			0.89			
PC aa C36:1	0.48	16.51	0.00	0.36	15.77	0.01	0.32	16.0	0.04	-0.74	-0.53	0.21
PC aa C36:2			0.28			0.21			0.62			
PC aa C36:3			0.41			0.11			0.71			
PC aa C36:4			0.24			0.12			0.89			
PC aa C36:5	0.77	16.23	0.00	0.73	14.96	0.00	0.62	15.3	0.00	-1.27	-0.91	0.35
PC aa C36:6	0.54	12.31	0.00			0.05			0.07			
PC aa C38:0	0.58	16.12	0.00	0.61	14.80	0.01	0.55	15.9	0.00	-1.32	-0.24	1.08
PC aa C38:3	0.55	15.31	0.00	0.63	13.91	0.01	0.47	15.0	0.00	-1.40	-0.29	1.10
PC aa C38:4	0.48	16.77	0.01	0.60	14.89	0.01	0.51	15.1	0.01	-1.88	-1.66	0.21
PC aa C38:5	0.68	15.85	0.00	0.75	14.03	0.00	0.52	14.9	0.00	-1.81	-0.94	0.87
PC aa C38:6			0.06	0.67	13.97	0.02	0.53	14.1	0.01			0.14
PC aa C40:2	0.44	17.01	0.00			0.07			0.06			
PC aa C40:3	0.57	16.61	0.00	0.63	14.80	0.01	0.41	16.6	0.04	-1.81	0.03	1.84
PC aa C40:4	0.55	15.64	0.00	0.56	13.86	0.01	0.53	14.3	0.00	-1.79	-1.32	0.47
PC aa C40:5	0.57	15.09	0.01	0.66	13.85	0.00	0.58	14.0	0.00	-1.24	-1.07	0.17
PC aa C40:6	0.53	14.78	0.01	0.72	13.65	0.00	0.60	13.7	0.00	-1.14	-1.13	0.01
PC aa C42:0	0.45	16.96	0.01	0.45	14.26	0.02	0.51	15.7	0.02	-2.70	-1.28	1.42
PC aa C42:1	0.46	15.85	0.03			0.11	0.46	15.4	0.01		-0.46	
PC aa C42:2	0.50	15.57	0.00	0.56	15.86	0.01			0.10	0.28		
PC aa C42:4	0.39	15.86	0.02			0.10	0.40	15.4	0.04		-0.48	
PC aa C42:5	0.67	15.51	0.00	0.60	14.22	0.01	0.58	15.3	0.00	-1.30	-0.25	1.04
PC aa C42:6	0.52	15.77	0.00	0.53	13.43	0.01	0.48	13.7	0.03	-2.34	-2.07	0.28
PC ae C30:0			0.25	0.44	9.10	0.02	0.45	10.3	0.00			1.22
PC ae C30:2			0.06			0.14	0.39	13.7	0.02			
PC ae C32:1	0.57	14.47	0.01	0.62	12.50	0.01	0.60	14.6	0.01	-1.98	0.08	2.06
PC ae C32:2	0.70	14.97	0.00	0.59	13.67	0.02	0.73	15.5	0.00	-1.30	0.57	1.87
PC ae C34:0			0.25	0.44	10.87	0.04	0.46	11.6	0.00			0.75
PC ae C34:1	0.66	12.99	0.00	0.70	11.58	0.01	0.63	13.3	0.00	-1.40	0.29	1.70

Metabolite	Day 1			Day 2			Day 3			Phase shift Δ φ (dec.h)		
	AMP	phase (dec. h)	p value	AMP	phase (dec. h)	p value	AMP	phase (dec. h)	p value	D1 vs. D2	D1 vs. D3	D2 vs. D3
PC ae C34:2	0.53	16.46	0.01	0.53	13.59	0.04	0.53	15.8	0.00	-2.87	-0.63	2.24
PC ae C34:3	0.83	17.00	0.00	0.65	14.70	0.01	0.88	16.8	0.00	-2.30	-0.15	2.14
PC ae C36:0	0.45	16.50	0.00			0.08	0.44	14.8	0.00	7.50		
PC ae C36:1	0.54	13.96	0.01	0.62	13.16	0.01	0.51	13.9	0.01	-0.80	-0.05	0.75
PC ae C36:2			0.39	0.45	14.64	0.04	0.49	16.0	0.01			1.34
PC ae C36:3	0.54	16.68	0.00	0.59	14.09	0.01	0.56	16.3	0.01	-2.58	-0.34	2.24
PC ae C36:4	0.71	16.43	0.00	0.61	14.20	0.00	0.56	15.5	0.00	-2.23	-0.95	1.27
PC ae C36:5	0.77	17.01	0.00	0.52	14.88	0.04	0.66	16.5	0.00	-2.13	-0.56	1.57
PC ae C38:0	0.63	15.45	0.00	0.64	14.47	0.00	0.65	14.6	0.00	-0.99	-0.88	0.11
PC ae C38:2			1.00			0.65	0.31	20.6	0.02			
PC ae C38:3	0.46	15.65	0.01	0.48	14.27	0.03	0.53	14.9	0.00	-1.38	-0.74	0.64
PC ae C38:4	0.53	15.55	0.00	0.56	13.97	0.01	0.51	15.8	0.00	-1.59	0.23	1.82
PC ae C38:5	0.61	16.22	0.00	0.53	14.40	0.02	0.54	15.9	0.01	-1.82	-0.28	1.55
PC ae C38:6	0.62	16.08	0.00	0.60	13.83	0.01	0.64	16.0	0.00	-2.25	-0.11	2.15
PC ae C40:1	0.41	19.03	0.04	0.42	18.75	0.01			0.05	-0.27		
PC ae C40:2			0.13	0.61	14.34	0.00	0.55	16.0	0.01			1.71
PC ae C40:3	0.42	16.00	0.02	0.53	13.93	0.00	0.48	15.9	0.03	-2.08	-0.08	2.00
PC ae C40:4	0.45	16.14	0.01	0.57	14.72	0.01	0.48	16.1	0.03	-1.42	0.01	1.43
PC ae C40:5	0.48	16.32	0.02	0.53	14.24	0.01	0.58	15.8	0.00	-2.07	-0.56	1.52
PC ae C40:6	0.52	15.44	0.01	0.65	13.63	0.01	0.65	14.9	0.00	-1.82	-0.56	1.26
PC ae C42:1	0.37	14.43	0.02			0.11			0.07			
PC ae C42:2	0.53	18.14	0.00	0.49	14.24	0.04	0.48	16.1	0.01	-3.90	-2.04	1.86
PC ae C42:3	0.44	16.04	0.02			0.06			0.06			
PC ae C42:4			0.07	0.49	14.19	0.03	0.53	15.4	0.00			1.18
PC ae C42:5	0.47	15.99	0.02	0.45	14.72	0.02	0.59	15.8	0.01	-1.28	-0.16	1.12
PC ae C44:3			0.18	0.36	15.48	0.04	0.50	17.1	0.00			1.59
PC ae C44:4			0.07	0.45	14.10	0.04			0.06			
PC ae C44:5	0.45	16.42	0.03	0.52	14.55	0.01	0.55	16.8	0.00	-1.87	0.36	2.23
PC ae C44:6	0.58	15.63	0.01	0.54	14.25	0.03	0.57	15.9	0.00	-1.38	0.26	1.64
SM (OH) C14:1	0.45	16.13	0.03	0.57	13.54	0.01	0.48	16.2	0.02	-2.59	0.07	2.67
SM (OH) C16:1			0.06	0.58	13.22	0.00	0.43	15.2	0.02			1.99
SM (OH) C22:1	0.50	16.73	0.03	0.59	14.16	0.00			0.10	-2.57		
SM (OH) C22:2	0.62	16.12	0.01	0.60	13.93	0.00	0.49	15.6	0.01	-2.19	-0.47	1.72
SM (OH) C24:1			0.19	0.38	12.87	0.01			0.51			
SM C16:0	0.58	16.80	0.00	0.54	13.39	0.00	0.51	16.3	0.01	-3.42	-0.49	2.93
SM C16:1			0.06	0.58	13.36	0.01	0.48	14.9	0.01			1.57
SM C18:0	0.60	15.00	0.01	0.56	13.15	0.02	0.44	15.3	0.04	-1.84	0.33	2.18
SM C18:1	0.57	15.15	0.02	0.58	12.88	0.00	0.53	14.9	0.01	-2.26	-0.27	1.99
SM C20:2	0.69	16.41	0.02			0.11	0.37	15.8	0.05		-0.64	
SM C24:0	0.50	16.34	0.03	0.54	13.97	0.00	0.41	16.7	0.04	-2.37	0.33	2.70
SM C24:1	0.49	16.15	0.02	0.43	14.44	0.01	0.44	15.9	0.05	-1.71	-0.23	1.48
SM C26:0	0.43	16.64	0.05	0.30	13.08	0.02			0.23	-3.56		
SM C26:1			0.08			0.12	0.41	15.3	0.01			

Supplementary Table S5. Metabolic profiling data: MetaCycle analysis (relative amplitude, acrophase) for each measured metabolite on 3 days (day1: sleep, day 2: sleep deprivation, day 3: recovery sleep), and the change in phase between each day.

Metabolite	Day 1			Day 2			Day 3			Phase shift Δ φ (dec.h)		
	r-AMP	Phase (dec. h)	FDR P	r-AMP	Phase (dec. h)	FDR P	r-AMP	Phase (dec. h)	FDR P	D1 vs. D2	D1 vs. D3	D2 vs. D3
Alanine			1.00			1.00			1.00			
Arginine			1.00			1.00			1.00			
Asparagine			0.16			0.67	0.13	22.86	0.01			
Citrulline			0.84			1.00			1.00			
Glutamine			1.00			1.00			1.00			
Glutamate	0.40	2.52	0.01			0.25			0.60			
Glycine	0.11	2.26	0.02			1.00			0.37			
Histidine			1.00			1.00			1.00			
Isoleucine			1.00			1.00			1.00			
Leucine			1.00			1.00			1.00			
Lysine			1.00			1.00			1.00			
Methionine			1.00			1.00			1.00			
Ornithine			1.00			1.00			1.00			
Phenylalanine			1.00			1.00			1.00			
Proline			1.00			1.00			1.00			
Threonine			1.00			1.00			0.24			
Tryptophan			1.00			1.00			1.00			
Tyrosine			1.00			1.00			1.00			
Valine			1.00			1.00			1.00			
Carnitine			1.00			1.00			1.00			
Acetylcarnitine			1.00			1.00			1.00			
Propionylcarnitine			1.00			1.00			1.00			
Butyrylcarnitine			1.00			1.00			0.76			
Valerylcarnitine			1.00			1.00			1.00			
Tetradecenoylcarnitine			1.00			1.00			1.00			
Hexadecanoylcarnitine			1.00			1.00			1.00			
Octadecanoylcarnitine			1.00			1.00			1.00			
Octadecenoylcarnitine			1.00			1.00			1.00			
Octadecadienylcarnitine			1.00			1.00			1.00			
ADMA			1.00			1.00			1.00			
alpha-AAA			1.00			0.86			1.00			
Kynurenine			1.00			1.00			0.60			
SDMA	0.03	9.97	0.00	0.03	12.04	0.01			0.05	2.07		
Serotonin			1.00			1.00			1.00			
t4-OH-Pro	0.34	2.16	0.00	0.40	2.71	0.00	0.38	2.40	0.00	0.55	0.24	-0.31
Taurine			1.00			1.00			1.00			
lysoPC a C16:0	0.21	20.12	0.00	0.18	19.16	0.00	0.16	20.49	0.02	-0.97	0.37	1.34
lysoPC a C16:1	0.19	19.61	0.00	0.17	18.16	0.00	0.15	19.71	0.01	-1.45	0.10	1.55
lysoPC a C17:0	0.18	21.06	0.00	0.14	19.35	0.00			0.60	-1.71		
lysoPC a C18:0	0.18	20.82	0.01	0.15	19.36	0.00			0.27	-1.46		
lysoPC a C18:1	0.23	20.32	0.00	0.20	20.44	0.00	0.19	20.98	0.00	0.12	0.66	0.53
lysoPC a C18:2	0.35	20.90	0.00	0.34	20.38	0.00	0.30	20.46	0.00	-0.51	-0.43	0.08
lysoPC a C20:3	0.24	20.26	0.00	0.20	19.72	0.00	0.22	19.76	0.00	-0.54	-0.49	0.04
lysoPC a C20:4	0.23	20.76	0.00	0.22	20.17	0.00	0.17	20.39	0.00	-0.58	-0.36	0.22

Metabolite	Day 1			Day 2			Day 3			Phase shift Δ φ (dec.h)		
	r-AMP	Phase (dec. h)	FDR P	r-AMP	Phase (dec. h)	FDR P	r-AMP	Phase (dec. h)	FDR P	D1 vs. D2	D1 vs. D3	D2 vs. D3
lysoPC a C24:0			1.00			1.00			1.00			
lysoPC a C26:0			1.00			1.00			1.00			
lysoPC a C26:1			1.00			1.00			1.00			
lysoPC a C28:0			1.00			1.00			1.00			
lysoPC a C28:1			1.00			1.00			1.00			
PC aa C28:1			1.00			1.00			1.00			
PC aa C30:0			0.11	0.12	6.29	0.00			0.06			
PC aa C32:0			0.76			1.00			1.00			
PC aa C32:1	0.20	11.90	0.00	0.16	10.96	0.00	0.17	10.78	0.00	-0.94	-1.11	-0.17
PC aa C32:3			1.00			1.00			1.00			
PC aa C34:1			1.00			1.00			1.00			
PC aa C34:2			1.00			1.00			1.00			
PC aa C34:3	0.13	14.29	0.01			1.00			1.00			
PC aa C34:4			0.17			1.00			1.00			
PC aa C36:0			1.00			1.00			1.00			
PC aa C36:1	0.08	17.88	0.04			1.00			1.00			
PC aa C36:2			1.00			1.00			1.00			
PC aa C36:3			1.00			1.00			1.00			
PC aa C36:4			1.00			1.00			1.00			
PC aa C36:5	0.11	16.18	0.00			0.05			0.60	7.82		
PC aa C36:6	0.08	13.18	0.02			1.00			1.00			
PC aa C38:0			1.00			1.00			1.00			
PC aa C38:3			1.00			0.41			1.00			
PC aa C38:4			1.00			1.00			1.00			
PC aa C38:5	0.11	16.00	0.01			0.32			1.00			
PC aa C38:6			0.58			0.09			1.00			
PC aa C40:2			1.00			1.00			1.00			
PC aa C40:3			1.00			1.00			1.00			
PC aa C40:4			0.06			1.00			1.00			
PC aa C40:5	0.26	15.33	0.03			1.00			1.00			
PC aa C40:6	0.13	15.03	0.01			0.11			1.00			
PC aa C42:0			1.00			1.00			1.00			
PC aa C42:1			1.00			1.00			1.00			
PC aa C42:2			1.00			1.00			1.00			
PC aa C42:4			1.00			1.00			1.00			
PC aa C42:5			0.13			0.74			1.00			
PC aa C42:6			0.68			1.00			1.00			
PC ae C30:0			0.35			1.00			1.00			
PC ae C30:2			1.00			1.00			1.00			
PC ae C32:1			0.21			1.00			1.00			
PC ae C32:2			0.91			1.00			0.07			
PC ae C34:0			0.90			1.00			1.00			
PC ae C34:1	0.12	14.90	0.04			0.35			1.00			

Metabolite	Day 1			Day 2			Day 3			Phase shift Δ φ (dec.h)		
	r-AMP	Phase (dec. h)	FDR P	r-AMP	Phase (dec. h)	FDR P	r-AMP	Phase (dec. h)	FDR P	D1 vs. D2	D1 vs. D3	D2 vs. D3
PC ae C34:2			0.68			1.00			1.00			
PC ae C34:3	0.07	17.81	0.03			1.00	0.10	18.00	0.00		0.19	
PC ae C36:0			1.00			1.00			1.00			
PC ae C36:1			0.53			1.00			1.00			
PC ae C36:2			1.00			1.00			1.00			
PC ae C36:3			0.55			1.00			0.76			
PC ae C36:4	0.10	16.45	0.03			1.00			1.00			
PC ae C36:5			0.11			1.00			0.60			
PC ae C38:0			0.27			1.00			1.00			
PC ae C38:2			1.00			1.00			1.00			
PC ae C38:3			0.41			1.00			1.00			
PC ae C38:4			1.00			1.00			1.00			
PC ae C38:5			0.76			1.00			0.99			
PC ae C38:6			0.68			1.00			0.63			
PC ae C40:1			1.00			1.00			1.00			
PC ae C40:2			1.00			1.00			1.00			
PC ae C40:3			1.00			1.00			1.00			
PC ae C40:4			1.00			1.00			1.00			
PC ae C40:5			0.92			1.00			1.00			
PC ae C40:6			0.91			1.00			1.00			
PC ae C42:1			1.00			1.00			1.00			
PC ae C42:2			0.21			1.00			1.00			
PC ae C42:3			1.00			1.00			1.00			
PC ae C42:4			1.00			1.00			1.00			
PC ae C42:5			0.55			1.00			1.00			
PC ae C44:3			1.00			1.00			1.00			
PC ae C44:4			1.00			1.00			1.00			
PC ae C44:5			1.00			1.00			1.00			
PC ae C44:6			0.56			1.00			1.00			
SM (OH) C14:1			1.00			1.00			0.64			
SM (OH) C16:1			1.00			1.00			1.00			
SM (OH) C22:1			1.00			0.65			1.00			
SM (OH) C22:2			1.00			1.00			0.60			
SM (OH) C24:1			0.87			1.00			1.00			
SM C16:0			1.00			1.00			0.08			
SM C16:1			1.00			1.00			1.00			
SM C18:0			1.00			1.00			1.00			
SM C18:1			1.00			1.00			1.00			
SM C20:2			1.00			1.00			1.00			
SM C24:0			1.00			1.00			1.00			
SM C24:1			1.00			1.00			1.00			
SM C26:0			1.00			1.00			1.00			
SM C26:1			1.00			1.00			1.00			

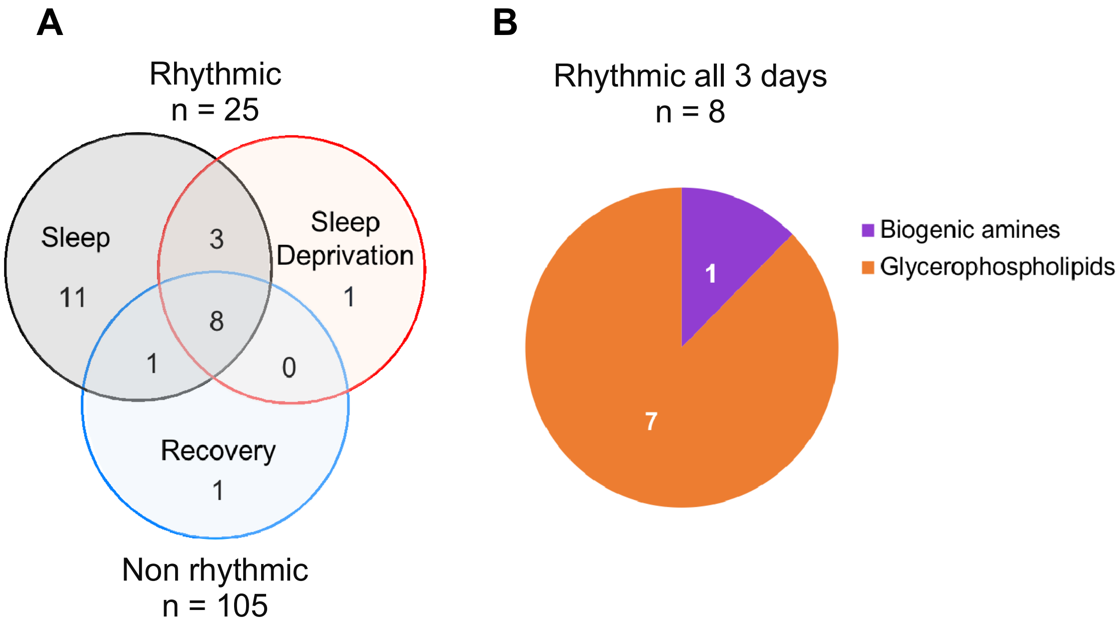

Supplementary Figure S3. Metabolites with a significant diurnal rhythm in the 3 study days with MetaCycle analysis. (A) Venn diagram showing the number of metabolites exhibiting a significant rhythmicity on day 1 (sleep, grey circle), day 2 (sleep deprivation, red circle), day 3 (recovery sleep, blue circle). (B) Pie charts showing the proportion of metabolites from each metabolite class exhibiting a significant diurnal rhythm all days.

Supplementary Table S6. Metabolic profiling of male data reanalysed using our new methods and stricter exclusion criteria: cosinor analysis (amplitude, acrophase) for each measured metabolite on day 1 (sleep) and day 2 (sleep deprivation) and the change in phase between day 1 and day 2.

Metabolite	Day 1			Day 2			Phase shift
	AMP	Phase (dec.h)	p-value	AMP	Phase (dec.h)	p-value	Δ φ (dec.h)
Alanine			0.27			0.19	
Arginine			0.32			0.11	
Asparagine			0.17			0.10	
Aspartate			0.11	0.45	23.04	0.00	
Citrulline			0.61			0.57	
Glutamine			0.10			0.06	
Glutamate	0.95	1.61	0.00	0.7	1.06	0.05	-0.55
Glycine			0.49			0.24	
Histidine			0.37			0.13	
Isoleucine			0.08	0.78	22.39	0.05	
Leucine			0.22			0.11	
Lysine			0.42			0.25	
Methionine			0.48			0.16	
Ornithine	0.48	23.1	0.03	0.73	22.78	0.02	-0.32
Phenylalanine			0.07			0.07	
Proline	0.67	21.06	0.02	0.89	21.06	0.01	0.00
Serine			0.14			0.19	
Threonine			0.68			0.18	
Tryptophan			0.26			0.45	
Tyrosine			0.13	0.71	21.45	0.05	
Valine			0.10	0.78	22.98	0.03	
Carnitine	0.46	5.92	0.02	0.52	6.08	0.01	0.16
Acetylcarnitine	0.74	13.68	0.02			0.29	
Propionylcarnitine			0.22	0.77	1.07	0.01	
Hydroxybutyrylcarnitine			0.07			0.05	
Butyrylcarnitine	0.71	2.93	0.00	0.79	3.11	0.00	0.18
Valerylcarnitine			0.61	0.55	1.13	0.02	
Hydroxyhexanoylcarnitine			0.42			0.98	
Hydroxyvalerylcarnitine			0.22			0.55	
Pimelylcarnitine	0.87	14.85	0.01			0.10	
Tetradecenoylcarnitine	0.68	13.41	0.01			0.10	
Hexadecanoylcarnitine	0.84	15.98	0.00	0.71	16.82	0.01	0.84
Hydroxyhexadecanoylcarnitine			0.18			0.19	
Octadecanoylcarnitine	0.36	14.97	0.02	0.53	17.32	0.03	2.35
Octadecenoylcarnitine	0.8	13.68	0.01	0.75	13.82	0.03	0.14
Octadecadienylcarnitine	0.83	14.57	0.00	0.8	14.11	0.00	-0.46
ADMA			0.39			0.28	
alpha-AAA	0.66	23.1	0.00	0.72	23.07	0.01	-0.03
Creatinine			0.95			0.29	
DOPA			0.79			0.18	
Kynurenine	0.56	11.83	0.02	0.54	9.74	0.02	-2.09
SDMA	0.51	12.88	0.00	0.46	14.15	0.01	1.27
Sarcosine	0.65	21.79	0.01	0.68	22.38	0.04	0.59
Serotonin			0.06			0.30	
DMA	0.37	11.56	0.02			0.07	
Taurine	0.66	15.77	0.00			0.18	
lysoPC a C16:0	0.6	16.02	0.00	0.74	16.86	0.00	0.84

Metabolite	Day 1			Day 2			Phase shift
	**AMP**	**Phase (dec.h)**	**p-value**	**AMP**	**Phase (dec.h)**	**p-value**	**Δ φ (dec.h)**
lysoPC a C16:1			0.25			0.11	
lysoPC a C17:0			0.18	0.58	17.4	0.04	
lysoPC a C18:0	0.62	15.95	0.00	0.69	16.87	0.00	0.92
lysoPC a C18:1			0.21			0.11	
lysoPC a C18:2	0.68	21.86	0.01	0.75	22.01	0.00	0.15
lysoPC a C20:3			0.05			0.06	
lysoPC a C20:4			0.06			0.11	
lysoPC a C24:0			0.14	0.49	17.55	0.03	
lysoPC a C26:0			0.14			0.11	
lysoPC a C26:1			0.58			0.07	
lysoPC a C28:0			0.24			0.12	
lysoPC a C28:1			0.11			0.13	
PC aa C24:0			0.24	0.54	18.56	0.03	
PC aa C28:1			0.13			0.10	
PC aa C30:0	0.67	1.71	0.00	0.79	23.96	0.00	-1.75
PC aa C32:0			0.06	0.57	20.63	0.00	
PC aa C32:1			0.58			0.07	
PC aa C32:3			0.97	0.49	21.71	0.04	
PC aa C34:1			0.08			0.30	
PC aa C34:2			0.10			0.28	
PC aa C34:3			0.31	0.59	21.19	0.00	
PC aa C34:4			0.58			0.34	
PC aa C36:0			0.32			0.39	
PC aa C36:1	0.43	15.19	0.04	0.43	20.05	0.03	4.86
PC aa C36:2			0.14			0.21	
PC aa C36:3	0.48	17.22	0.01	0.54	19.97	0.02	2.75
PC aa C36:4	0.54	17.04	0.01			0.05	
PC aa C36:5	0.5	17.07	0.03	0.51	19.66	0.02	2.59
PC aa C36:6			0.16			0.07	
PC aa C38:0	0.71	14.99	0.00	0.49	15.36	0.01	0.37
PC aa C38:3	0.71	15.77	0.00	0.64	17.22	0.01	1.45
PC aa C38:4	0.76	16.16	0.00	0.61	17.7	0.01	1.54
PC aa C38:5	0.69	15.86	0.00	0.56	17.45	0.02	1.59
PC aa C38:6	0.74	15.94	0.00	0.71	17.19	0.00	1.25
PC aa C40:2	0.6	14.43	0.00	0.6	16.99	0.01	2.56
PC aa C40:3	0.7	15.42	0.00	0.52	16.71	0.00	1.29
PC aa C40:4	0.76	15.41	0.00	0.59	16.66	0.02	1.25
PC aa C40:5	0.8	15.62	0.00	0.64	16.41	0.00	0.79
PC aa C40:6	0.81	15.62	0.00	0.73	17.03	0.00	1.41
PC aa C42:0	0.72	14.87	0.00	0.55	16.04	0.01	1.17
PC aa C42:1	0.6	15.24	0.00	0.54	16.61	0.03	1.37
PC aa C42:2	0.62	15.01	0.00	0.62	17.32	0.01	2.31
PC aa C42:4	0.68	15.22	0.00	0.59	16.77	0.03	1.55
PC aa C42:5	0.76	15.25	0.00	0.67	16.81	0.01	1.56
PC aa C42:6	0.68	15.22	0.00			0.06	
PC ae C30:0			0.21	0.52	23.14	0.02	
PC ae C30:2			0.15			0.07	

Metabolite	Day 1			Day 2			Phase shift
	AMP	Phase (dec.h)	p-value	AMP	Phase (dec.h)	p-value	Δ φ (dec.h)
PC ae C32:1	0.53	14.31	0.01			0.13	
PC ae C32:2	0.57	14.23	0.01	0.49	17.83	0.02	3.60
PC ae C34:0			0.46			0.21	
PC ae C34:1	0.37	12.95	0.12			0.32	
PC ae C34:2	0.46	15.78	0.02			0.06	
PC ae C34:3	0.84	15.53	0.00	0.61	16.35	0.01	0.82
PC ae C36:0	0.49	14.6	0.01			0.17	
PC ae C36:1			0.29			0.31	
PC ae C36:2			0.93	0.44	20.14	0.04	
PC ae C36:3	0.58	15.25	0.00			0.06	
PC ae C36:4	0.76	14.99	0.00	0.49	15.45	0.04	0.46
PC ae C36:5	0.8	15.01	0.00	0.52	14.27	0.02	-0.74
PC ae C38:0	0.53	16.43	0.01	0.58	18.92	0.01	2.49
PC ae C38:2	0.34	2.06	0.02	0.58	20.26	0.00	-5.80
PC ae C38:3			0.08			0.14	
PC ae C38:4	0.61	15.06	0.00			0.07	
PC ae C38:5	0.74	15.03	0.00	0.54	15.73	0.03	0.70
PC ae C38:6	0.74	15	0.00	0.54	15.83	0.03	0.83
PC ae C40:1			0.22	0.61	20.2	0.01	
PC ae C40:2			0.07			0.24	
PC ae C40:3	0.52	14.37	0.01	0.42	16.29	0.02	1.92
PC ae C40:4	0.61	15.32	0.00	0.44	16.19	0.04	0.87
PC ae C40:5	0.66	15.03	0.00	0.54	16.15	0.03	1.12
PC ae C40:6	0.62	14.74	0.00	0.59	16.24	0.01	1.50
PC ae C42:1	0.48	14.64	0.04	0.49	18.1	0.05	3.46
PC ae C42:2	0.47	14.81	0.03			0.06	
PC ae C42:3			0.10	0.37	18.41	0.03	
PC ae C42:4	0.64	15.21	0.00	0.56	16.83	0.02	1.62
PC ae C42:5	0.65	15.06	0.00	0.57	15.96	0.02	0.90
PC ae C44:3			0.07	0.46	19.11	0.03	
PC ae C44:4	0.6	15.02	0.00	0.53	16.54	0.04	1.52
PC ae C44:5	0.69	15.19	0.00	0.59	16.06	0.01	0.87
PC ae C44:6	0.7	15.09	0.00	0.58	16.02	0.02	0.93
SM (OH) C14:1			0.07			0.25	
SM (OH) C16:1	0.43	13.36	0.05			0.28	
SM (OH) C22:1			0.11			0.31	
SM (OH) C22:2	0.45	14.36	0.03			0.19	
SM (OH) C24:1	0.39	12.31	0.02			0.34	
SM C16:0	0.55	14.72	0.01			0.07	
SM C16:1	0.58	14.48	0.01			0.06	
SM C18:0	0.48	14.69	0.02			0.09	
SM C18:1	0.51	14.34	0.01			0.07	
SM C20:2			0.12			0.16	
SM C24:0	0.41	14.78	0.04			0.12	
SM C24:1	0.49	14.99	0.01			0.07	
SM C26:0			0.06	0.42	15.14	0.05	
SM C26:1	0.37	14.66	0.03			0.25	

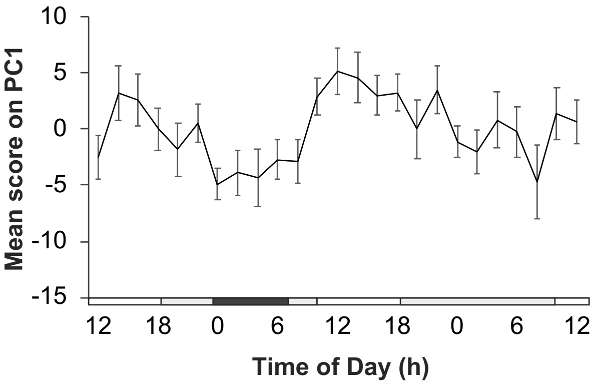

Supplementary Figure S4. PCA of male metabolites data (reanalysed using our new methods and stricter exclusion criteria) were carried out, and the change in mean score (± SEM) across all subjects on PC1 with time is shown. The black bar indicates the sleep period, 0 lux, supine; grey bars, wake periods, semi-recumbent position, <8 lux; white bars, awake and free movement, 90 lux.

Supplementary Table S7. Common metabolites to both sexes that exhibited significant diurnal rhythms with cosinor analysis on day 1 (sleep) and day 2 (sleep deprivation), and metabolites only rhythmic in males and only rhythmic in females, the acrophase times and the change in phase (Δ φ) between day 1 and day 2.

Metabolite	Male			Female		
	Phase (dec. h)		Phase shift	Phase (dec. h)		Phase shift
	day 1	day2	Δ φ (dec.h)	day 1	day2	Δ φ (dec.h)
Glutamate	1.61	1.06	-0.55	2.46	2.42	-0.04
alpha-AAA	23.10	23.07	-0.03	23.67	22.89	-0.78
SDMA	12.88	14.15	1.27	9.56	11.64	2.08
lysoPC a C16:0	16.02	16.86	0.84	20.28	19.48	-0.80
lysoPC a C18:0	15.95	16.87	0.92	20.68	20.03	-0.65
lysoPC a C18:2	21.86	22.01	0.15	20.38	20.21	-0.17
PC aa C30:0	1.71	23.96	-1.75	6.84	5.78	-1.06
PC aa C36:1	15.19	20.05	4.86	16.55	15.71	-0.84
PC aa C36:5	17.07	19.66	2.59	16.23	15.01	-1.22
PC aa C38:0	14.99	15.36	0.37	16.12	14.88	-1.24
PC aa C38:3	15.77	17.22	1.45	15.31	13.99	-1.32
PC aa C38:4	16.16	17.70	1.54	16.77	14.93	-1.84
PC aa C38:5	15.86	17.45	1.59	15.84	14.10	-1.74
PC aa C40:3	15.42	16.71	1.29	16.60	14.81	-1.79
PC aa C40:4	15.41	16.66	1.25	15.65	13.91	-1.74
PC aa C40:5	15.62	16.41	0.79	15.11	13.92	-1.19
PC aa C40:6	15.62	17.03	1.41	14.79	13.72	-1.07
PC aa C42:0	14.87	16.04	1.17	16.97	14.32	-2.65
PC aa C42:2	15.01	17.32	2.31	15.58	15.99	0.41
PC aa C42:5	15.25	16.81	1.56	15.52	14.27	-1.25
PC ae C32:2	14.23	17.83	3.60	15.13	13.58	-1.55
PC ae C34:3	15.53	16.35	0.82	17.27	14.70	-2.57
PC ae C36:4	14.99	15.45	0.46	16.72	14.27	-2.45
PC ae C38:0	16.43	18.92	2.49	15.46	14.58	-0.88
PC ae C38:5	15.03	15.73	0.70	16.63	14.48	-2.15
PC ae C38:6	15.00	15.83	0.83	16.44	13.88	-2.56
PC ae C40:3	14.37	16.29	1.92	16.00	14.06	-1.94
PC ae C40:4	15.32	16.19	0.87	16.69	14.88	-1.81
PC ae C40:5	15.03	16.15	1.12	16.31	14.37	-1.94
PC ae C40:6	14.74	16.24	1.50	15.45	13.71	-1.74
PC ae C44:5	15.19	16.06	0.87	17.03	14.64	-2.39
PC ae C44:6	15.09	16.02	0.93	15.63	14.23	-1.40
Ornithine	23.1	22.78	-0.32			
Proline	21.06	21.06	0.00			
Kynurenine	11.83	9.74	-2.09			
Sarcosine*	21.79	22.38	0.59			
Carnitine	5.92	6.08	0.16			
Butyrylcarnitine	2.93	3.11	0.18			
Hexadecanoylcarnitine	15.98	16.82	0.84			
Octadecanoylcarnitine	14.97	17.32	2.35			
Octadecenoylcarnitine	13.68	13.82	0.14			
Octadecadienylcarnitine	14.57	14.11	-0.46			
PC aa C36:3	17.22	19.97	2.75			
PC aa C42:1	15.24	16.61	1.37			
PC ae C38:2	2.06	20.26	-5.80			
PC ae C42:4	15.21	16.83	1.62			
PC ae C44:4	15.02	16.54	1.52			

Metabolite	Male			Female		
	Phase (dec. h)		Phase shift	Phase (dec. h)		Phase shift
	day 1	day2	Δ φ (dec.h)	day 1	day2	Δ φ (dec.h)
Asparagine				23.04	21.55	-1.49
t4-OH-Pro**				1.88	1.43	-0.45
lysoPC a C16:1				19.23	18.51	-0.72
lysoPC a C18:1				20.53	20.50	-0.03
lysoPC a C20:3				20.03	19.67	-0.36
lysoPC a C20:4				20.43	20.01	-0.42
PC aa C28:1				16.34	14.36	-1.98
PC aa C32:1				10.92	10.17	-0.75
PC aa C36:6				12.29	11.56	-0.73
PC ae C36:1				14.22	13.29	-0.93
PC ae C38:3				16.28	14.39	-1.89
SM (OH) C14:1				16.13	13.54	-2.59
SM (OH) C22:1				16.74	14.13	-2.61

* metabolites quantified only in males; ** metabolites quantified only in females

Supplementary Table S8. Percent change in metabolite concentrations (µM, mean ± SEM, 0:00 – 6:00 h) that were significantly different during sleep deprivation compared with baseline sleep in females and males.

Metabolite	Female			Male†		
	% change (mean ± SEM)					
PC aa C36:5	-14.2	±	2.4			
Glutamate	-9.9	±	4.7			
Lysine	-7.5	±	2.2			
PC aa C40:3	-7.2	±	2.8			
lysoPC a C18:0	-7.1	±	0.9			
Carnitine	-6.9	±	3.0			
SDMA	-6.4	±	1.5			
PC aa C38:5	-6.3	±	1.7			
PC ae C36:3	-6.0	±	2.4			
PC ae C42:2	-5.9	±	2.1			
Citrulline	-5.7	±	1.6			
PC aa C40:5	-5.5	±	0.6			
Histidine	-5.3	±	1.5			
PC aa C38:0	-4.7	±	2.2			
Threonine	3.9	±	1.5			
Hydroxyhexadecanoylcarnitine*				6.7	±	3.1
Tetradecanoylcarnitine*				10.4	±	4.1
SM (OH) C22:2				10.7	±	4.1
lysoPC a C18:1				11.0	±	4.7
Octanoylcarnitine*				11.9	±	3.9
PC ae C40:5				12.0	±	5.1
PC aa C32:3				12.1	±	5.0
PC ae C40:3				12.2	±	4.7
PC ae C40:6				12.2	±	5.3
SM (OH) C14:1				12.3	±	4.4
SM C20:2				12.3	±	4.8
PC aa C28:1				13.0	±	5.3
PC ae C38:4				13.5	±	5.3
PC aa C32:0				14.6	±	6.3
Propionylcarnitine				14.9	±	5.4
PC aa C36:6				15.1	±	5.8
Valerylcarnitine				15.5	±	5.1
PC ae C32:1				15.9	±	5.8
SM (OH) C16:1				15.9	±	5.0
PC ae C36:0				16.2	±	6.7
PC ae C40:2				16.6	±	5.7
lysoPC a C16:1				17.1	±	5.3
PC aa C34:4				17.1	±	5.3
PC ae C36:2				18.5	±	6.2
Tryptophan				19.6	±	6.0
PC ae C38:2				20.2	±	10.6
PC ae C34:1				20.7	±	6.5
PC ae C38:3				20.8	±	6.3
Taurine				21.0	±	5.7
Pimelylcarnitine*				21.5	±	9.1
PC ae C36:1				21.8	±	6.0
lysoPC a C17:0				22.0	±	6.4
PC aa C32:1				22.2	±	6.4
PC aa C30:0				25.1	±	7.2
PC ae C30:0				25.4	±	5.8
PC ae C34:0				28.6	±	7.3
Serotonin*				43.6	±	20.1

† Data taken directly from Davies *et al.,* 2014; * metabolites quantified only in males
