# Supplementary material for: Effect of acute total sleep deprivation on plasma melatonin, cortisol and metabolite rhythms in females
Source: Eur J Neurosci. 2019 May 2;51(1):366–78. doi: 10.1111/ejn.14411 (PMC7027445; doi:10.1111/ejn.14411)
Supplement: Supplementary file 2 [file EJN-51-366-s002.docx]

| Effect of Acute Total Sleep Deprivation on Plasma Melatonin, Cortisol and Metabolite Rhythms in Females | |
| --- | --- |
|  | [Honma, Aya](javascript:popWindow('ejn?PARAMS=xik_45skVeRgoSsGSn4rRKBsFfadMHB3AtDgQ2ECDxVs4W9weqqApuuWFz598QiJhvQGYZ7LfGSmQWtiNgkxt7obTtqZqzFvgtUJMgsrT5sRoFAxyojzkoP91cHsThsXiLVvAsn2PPj8vSDu8Ktgb36X4HSt64JECsCEfXchGJmZcXeecGh','mailpopup_1599',%20900,%20775);) ;Revell, Victoria; Gunn, Pippa; Davies, Sarah; Middleton, Benita; Raynaud, Florence; Skene, Debra |

_____________________________________________________________________________________

| Review timeline: | Submission date: 04/01/19  Editorial Decision: Minor Revision  Revision Received: 21/02/19  Accepted: 12/03/19 |  |
| --- | --- | --- |
|  |  |  |

Editor: Rae Silver

Reviewer 1: Karen Gamble

Reviewer 2: Ari Shechter

_____________________________________________________________________________________

1st Editorial Decision	11/02/19

_____________________________________________________________________________________

Effect of Acute Total Sleep Deprivation on Plasma Melatonin, Cortisol and Metabolite Rhythms in Females 

Dear Dr. Honma, 

Thank you for your submission to the special issue of EJN on Circadian Rhythms. It has now been was reviewed by two external reviewers as well as ourselves.  As you will note, the reviewers (and we) really appreciate your work and think it will be a great contribution to the special issue.  They have raised a few minor points  that we think can be readily be made to improve the paper. Please carefully address each of the points that they have raised a a revised version of the manuscript. 

In addition, please note the following points that will need to be addressed in the revised version. 
- Please be more forthright about the ethical approval for the study. 
- The figures need to be of a higher quality, 300 -600 dpi.   (And not in pdf format). 
- Please include a graphic abstract and text. 
- The concentrations of melatonin and cortisol are expressed in different ways (pg/ml   and nanomol/L respectively). Is this the normal convention?   
- Figures 3 B and C and S2 B:  maybe give the why 'X' axis some sort of label.  Perhaps  'molecular species'   

When revising the manuscript, please embolden or underline major changes to the text so they are easily identifiable and please don't leave 'track change' formatting marks in your paper.  Please ensure that you provide a text and a figure file for the Graphical Abstract (as detailed in the instructions below).  When carrying out your revisions please refer to the checklist below and visit the EJN author guidelines at www.ejneuroscience.org 

When finalized, please upload your complete revised manuscript onto the website, as a Word file (.doc, or .docx).  Please also ensure that a complete set of tables and figures is included as separate files, even if these have not changed from the originals.  At this stage it is necessary to provide high resolution figures.  Please see important instructions below. 

Please go into https://mc.manuscriptcentral.com/ejn - Author Centre - manuscripts with decisions where you will find a 'create a revision' link under 'actions'. We ask that you please indicate the way in which you have responded to the points raised by the Editors and Reviewers in a letter. Please upload this response letter as a separate Word (.doc xor PDF) file using the file designation "Authors' Response to Reviewers" when uploading your manuscript files.  Please DO NOT submit your revised manuscript as a new one.  Also, please note that only the  Author who submitted the original version of the manuscript should submit a revised version. 

If you are able to respond fully to the points raised, we would be pleased to receive a revision of your paper within 30 days. 

Thank you again for your support of this Special Issue of EJN. 

Best wishes, 

Rae Silver 
Section Editor and Special Issue Editor 

and 

Paul Bolam & John Foxe 
co-Editors in Chief, EJN 


Reviews: 

Reviewer: 1 

This is an exciting report of circadian and sleep modulating effects on peripheral metabolites in women, Authors are commended for their sophisticated analytical techniques and rigorous experimental design. The manuscript is well written, and these results should be of great interest to the neuroscience community. Only a few minor comments are suggested to be addressed: 1. Abstract should mention lighting conditions. 2. Authors should comment on the regular cycle of feeding/fasting that may have contributed to the 24-hour rhythmicity of metabolites. For metabolite rhythmicity, what is the greatest zeitgeber – SCN input, environmental, light/dark, hormonal circulation, nutritional? 3. Somewhere in the manuscript, the multi-synaptic pathway regulating pineal melatonin rhythmicity and suppression by light should be described. The review appreciates the explanation of the influence of sleep deprivation on GIT-produced melatonin, but another explanation is that sleep deprivation influences SCN output. For example, sleep deprivation reduces SCN firing rate to ~60% (PMID: 17425221) in rats. 4. In Fig. 5, it may be helpful to label datapoints that greatly deviate from the diagonal. 5. It may be interesting to see a supplementary Table of metabolites that were rhythmic for females but not male and vice versa.

Reviewer: 2 

Comments to the Author 
Paper uses Liquid chromatography–mass spectrometry (LC/MS) metabolomics analysis to look at variation of metabolites across circadian phase under conditions of sleep deprivation, as well as measures of central circadian clock in females. 

Corrections for multiple comparison s appear sufficient (and definitely should be done considering the large number of metabolites investigates). Perhaps a little more details on the technique used (Benjamini-Hochberg false discovery rate) is warranted in the Data Analysis section considering the importance. 

Authors essentially control for menstrual cycle by including women on hormonal contraceptives and studying them only during the active phase. However, it would be important to know how these metabolites vary across menstrual cycle in ovulating women and whether sleep deprivation affects the variation differentially in ovulating women. This is similar comment concerning the sex based differences. To get at the sex differences it would be better to compares follicular, luteal phases in ovulating women vs. men. 

During the sleep deprivation period, participants are also in fasting state. Could this extended period of fasting contribute to differences in metabolite circulation? 

Table with comparison of the metabolites that were a) rhythmic and b) changed as a function of sleep deprivation, between men and women would be helpful. ____________________________________________________________________________________

Authors’ Response

_____________________________________________________________________________________

Dear Professor Silver, 

Thank you very much for accepting our manuscript for the special issue of EJN on Circadian Rhythms. It is a great honour that we could join this special issue as chronobiologists. 
We really appreciate your kind consideration. 

With best wishes, 
Aya Honma
